# Selenium nutritional status and cardiovascular diseases: mechanisms of cardioprotection and 21 years of global research trends in precision prevention (2005–2026)

**DOI:** 10.3389/fnut.2026.1920059

**Published:** 2026-08-31

**Authors:** Hao Chen, Shenghui Jin, Xinyi Yuan, Xuyang Zhong, Junqi Xiao, Jiayou Liao, Xiangtai Zeng

**Affiliations:** 1 Longnan Hospital, First Affiliated Hospital of Gannan Medical University, Ganzhou, Jiangxi, China; 2 Department of Clinical Medicine, First Clinical College, Gannan Medical University, Ganzhou, Jiangxi, China; 3 Department of Vascular Surgery, The First Affiliated Hospital of Gannan Medical University, Ganzhou, Jiangxi, China; 4 Department of Thyroid and Hernia Surgery, First Affiliated Hospital of Gannan Medical University, Ganzhou, Jiangxi, China

**Keywords:** bibliometric analysis, cardiovascular disease, ferroptosis, precision nutrition, selenium nutritional status, selenoprotein P, supplementation

## Abstract

**Background:**

Selenium contributes to selenoprotein-dependent redox regulation, thyroid hormone metabolism, and cellular defense, but its role in cardiovascular disease remains clinically uncertain. Observational studies have associated low or atypical selenium status with adverse cardiovascular outcomes, whereas supplementation trials have generally produced neutral or inconclusive results. This study mapped the development of selenium–cardiovascular research and integrated the identified themes with a clinically oriented narrative synthesis of selenium exposure, functional biomarkers, cardiovascular phenotypes, supplementation, and safety.

**Methods:**

Publications indexed in the Web of Science Core Collection, Scopus, PubMed, and Embase between January 1, 2005 and May 12, 2026 were retrieved. After document-type filtering, deduplication, and relevance screening, 1,133 documents were included. Publication trends, geographic and collaboration structures, journal relationships, co-cited references, keyword networks, thematic evolution, and citation bursts were analyzed using R, VOSviewer, CiteSpace, Pajek, Gephi, and Python. A descriptive design-level appraisal of the ten most locally cited documents complemented the bibliometric analyses.

**Results:**

Publication output increased substantially after 2015. Earlier research emphasized selenium deficiency, Keshan disease, glutathione peroxidase activity, and antioxidant defense, whereas more recent publications increasingly addressed cardiovascular mortality, heart failure, selenoprotein P, GPX4, ferroptosis, selenium nanoparticles, environmental co-exposures, and machine learning. The evidence base showed biological coherence but translational fragmentation: mechanistic studies supported plausible selenium-sensitive pathways, observational studies identified context-dependent risk associations, and randomized evidence did not demonstrate a broadly applicable cardiovascular benefit of supplementation. Major translational challenges included inadequate stratification by baseline selenium status, inconsistent distinction among dietary intake, total circulating selenium, and functional biomarkers, heterogeneous cardiovascular endpoints, geographic variation, and insufficient consideration of nonlinear dose–response and safety relationships.

**Conclusion:**

Current evidence does not support universal selenium supplementation for cardiovascular prevention. A more informative research strategy is to evaluate selenium status as a context-dependent marker of nutritional and functional vulnerability and to test targeted correction in biomarker-enriched populations defined by baseline status, cardiovascular phenotype, regional nutritional context, and a prespecified safety range.

## 1 Introduction

Cardiovascular diseases (CVDs) remain the leading cause of death worldwide. The World Health Organization estimated that in 2022, CVDs were responsible for 19.8 million deaths, representing about 32% of global mortality (1). Contemporary burden analyses also show substantial variation by geography, income, age, and risk-factor exposure (2). Established strategies targeting blood pressure, lipids, tobacco use, glycaemic control, physical inactivity, and dietary quality are central to prevention, but the continuing burden has sustained interest in nutritional factors that may modify cardiometabolic vulnerability.

Selenium is an essential trace element obtained primarily from food and incorporated as selenocysteine into 25 human selenoproteins (3–7, 110–112). These proteins include glutathione peroxidases, thioredoxin reductases, iodothyronine deiodinases, and selenoprotein P (SELENOP), which contribute to peroxide metabolism, redox signaling, thyroid hormone regulation, and selenium transport (5, 8–10, 109). Cardiovascular reviews have therefore focused on endothelial injury, inflammatory signaling, mitochondrial stress, lipid peroxidation, and myocardial dysfunction as biologically plausible selenium-sensitive processes (8, 11, 114, 115). However, five related but non-equivalent constructs need to be distinguished: selenium intake; total selenium status, measured in serum, plasma, whole blood, hair or nails; functional biomarkers of selenium status, such as SELENOP and glutathione peroxidase activity; selenium supplementation; and specific cardiovascular endpoints.

Selenium exposure is strongly shaped by the soil–plant-food chain. Soil selenium, agricultural conditions, food origin, dietary patterns, fortification, and supplement use differ across regions and produce substantial variation in intake and circulating concentrations (12–15). Climate-related changes may further reduce selenium availability in vulnerable areas (12). Therefore, an association in a low selenium population cannot be extrapolated to selenium-replete settings, and a circulating selenium concentration should not be interpreted without consideration of the biomarker, sampling context, inflammatory status, renal function and dietary background.

The best historical example of a context-dependent cardiac manifestation of severe Se deficiency is Keshan disease. This endemic cardiomyopathy was observed in selenium-poor areas of China but its aetiology is multifactorial and cannot be reduced to selenium deficiency alone (16–18). Long-term supplementation studies in chronic Keshan disease support clinical relevance within this specific deficiency-associated setting, while offering limited justification for extrapolation to the general population (19). The historical evidence therefore establishes cardiac relevance at the deficiency extreme rather than a universal rule that higher selenium exposure is preferable.

Human epidemiological evidence is more heterogeneous. Meta-analyses and cohort studies have reported inverse, null, and nonlinear associations between selenium biomarkers and coronary disease, cardiovascular mortality, and composite CVD outcomes (20–24). On the other hand, systematic reviews of randomised trials have not demonstrated consistent cardiovascular prevention with selenium supplementation, especially in populations without documented deficiency (25–27). Dose–response meta-analyses also suggest that effects on blood pressure, lipids, inflammation, diabetes risk, and mortality depend on baseline status, dose, formulation, population characteristics, and outcome definition (21, 28–30). These findings pose a translational problem in that mechanistic plausibility and observational risk associations do not necessarily translate to benefit with widespread supplementation.

Recent research has moved toward more specific phenotypes and biomarkers. Reviews have linked selenoprotein biology to cardiovascular stress and deficiency-related myocardial dysfunction (31–33). In heart failure, low selenium or low SELENOP has been associated with poorer functional capacity, incident disease, mortality, and immunoregulatory signatures (34–39, 113). In otherwise healthy adults, the distribution of selenium between GPx3 and SELENOP has shown distinct associations with cardiovascular risk factors (40). Studies of plasma selenium, dietary selenium, stroke, and composite CVD have likewise produced exposure- and endpoint-specific findings (41–45). These studies refine hypotheses of risk stratification, but their predominantly observational designs do not establish that supplementation improves clinical outcomes.

Bibliometric analysis can elucidate the emergence of these streams of evidence, the publications and research communities that became influential, and the thematic gaps that remain. Bibliometrix, VOSviewer and CiteSpace offer complementary analyses of scientific production, collaboration, co-citation, bibliographic coupling, clustering and temporal bursts (46–48). Methodological guidance emphasizes transparent data sources, reproducible search strategies, explicit thresholds, and interpretation that does not equate citation activity with evidence quality (49, 50).

Thus, in this study, we combined bibliometric mapping with a systematic, clinically-oriented synthesis of the selenium and CVD research published between 2005 and May 12, 2026. The goals were to reconstruct the intellectual and thematic development of the field, to identify the transition from deficiency and antioxidant research to functional biomarkers and precision-oriented questions, and to explain why biologically credible mechanisms have not been translated into a general recommendation for selenium supplementation. This analysis was designed to generate testable questions about the right population, biomarker, selenium form, dose, endpoint and safety window, as opposed to inferring treatment efficacy from bibliometric prominence.

## 2 Methods

### 2.1 Data sources and search strategy

We developed a comprehensive retrieval framework, using the four major databases, Web of Science Core Collection (WoSCC), Scopus, PubMed, and Embase, to map the evolving paradigms and resolve the ongoing controversies regarding selenium in cardiovascular health. Among these, the Science Citation Index Expanded (SCI-EXPANDED) was chosen as the specific data source for WoSCC. This multi-database integration strategy minimizes coverage blind spots, balances the evidence base, and increases the accuracy of subsequent bibliometric mapping.

We have built a dedicated search matrix. For the cardiovascular domain, we employed a large string of relevant terms (“cardiovascular*” OR “cardiac*” OR “cardio*” OR “heart*” OR “myocardial*” OR “pericardial*” OR “arrhythmia*” OR “artery disease*” OR “hypertension”) On the other hand, we used a simplified approach for the other subject term. We set “Selenium” as the sole keyword since it exhibits linguistic standardization. We skipped its elemental abbreviation (“Se”) in order not to have a flood of false positive noise. In PubMed the search was limited to titles and abstracts to increase the thematic relevance, in the other databases it was limited to titles, abstracts and author keywords. This limitation avoids interference by expanded keywords that diverge from the search objective and ensures network clarity. All searches and data extraction were completed May 12, 2026. The precise syntax for each database is listed in Supplementary Table 1.

**Figure 1:**
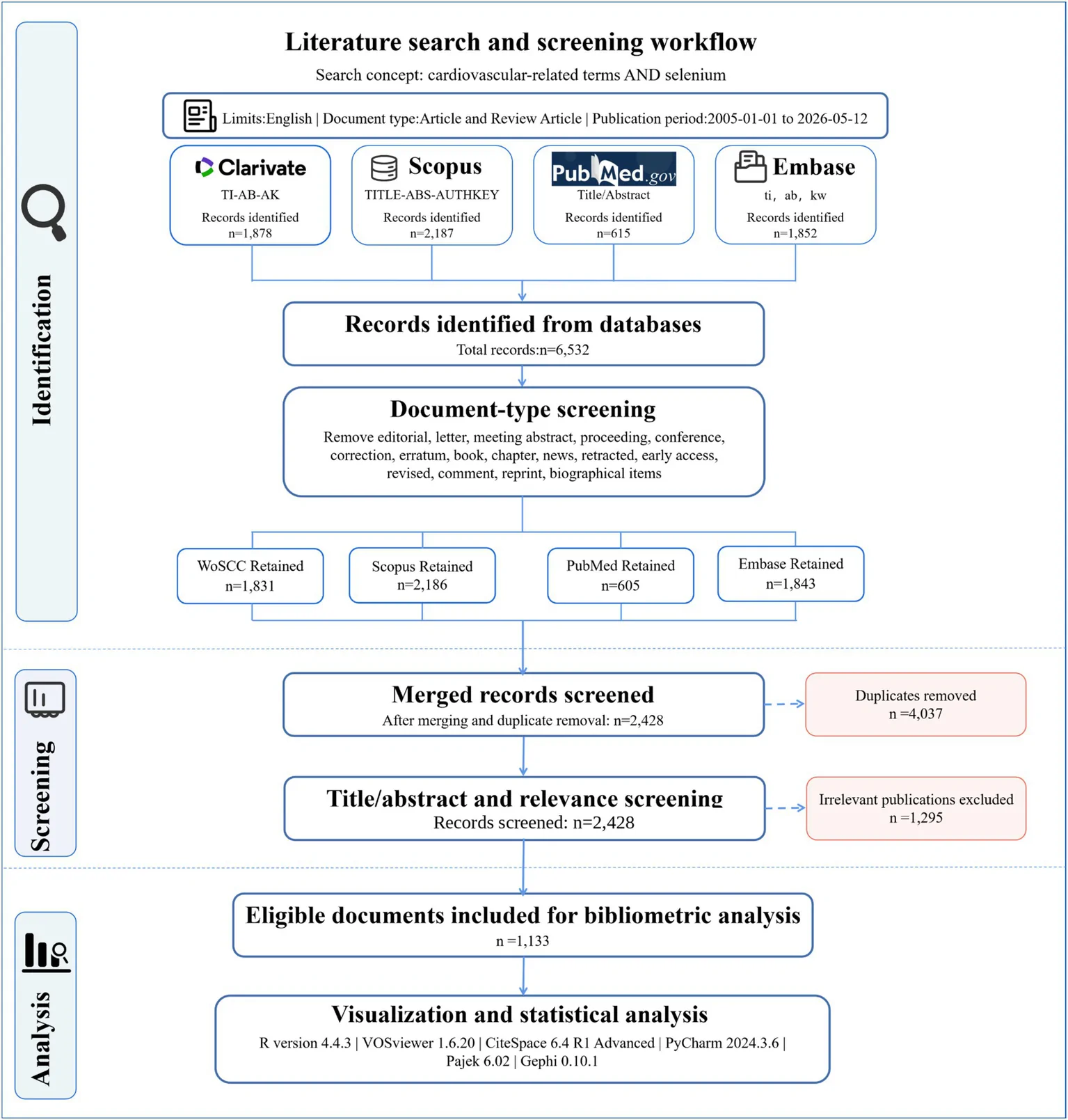
Literature search and screening workflow. Records were identified in the Web of Science Core Collection, Scopus, PubMed, and Embase. Document-type filtering retained 6,465 records; merging and exact duplicate removal yielded 2,428 unique records. Title/abstract and relevance screening excluded 1,295 records, leaving 1,133 documents for bibliometric analysis.

The time window was from January 1, 2005 to May 12, 2026. We restricted our inclusion to original articles and reviews in English language. The first data sweep resulted in 6,532 raw records (Scopus: 2,187; WoSCC: 1,878; Embase: 1,852; PubMed: 615). WoSCC data were exported and saved as plain text files with full records and cited references. The data from Scopus and Embase were exported in CSV format, whereas the data from PubMed were exported in NBIB format. First, the records from each database were passed through R individually to systematically remove non-target document types. This automated sweep removed editorials, letters, meeting abstracts, proceedings, conferences, corrections, errata, books, book chapters, news items, retracted papers, early access materials, revised manuscripts, comments, reprints and biographical items. We then merged the datasets in R, exactly deduplicated the datasets and standardized the data format for ease of further correlation analyses. This resulted in a refined set of 2,428 unique publications. Two independent investigators (Shenghui Jin and Hao Chen) screened the titles and abstracts for relevance to the topic. A third arbitrator (Xuyang Zhong) resolved deadlocks via active discussion. Full-text reviews were conducted when necessary; however, due to the large dataset, exact counts for specific exclusion reasons were not recorded. The filtering process removed irrelevant noise and yielded a corpus of 1,133 core documents for the final analysis. The exact procedure is illustrated in Figure 1.

### 2.2 Data analysis and visualization strategies

Six analytical tools were used to dissect the knowledge landscape from macro-trends to micro-topologies, including R, VOSviewer, CiteSpace, Pajek, Gephi, and Python.

#### 2.2.1 R software

We outlined the longitudinal publication trajectories across the four databases using Base R. Next, we calculated the real annual number of publications for the screened literature and a distribution heatmap of keywords relative to their own publication lifespan. The bibliometrix R-package also calculated macro-level geographic measures at the same time, making national scientific output, world collaboration mapping and other similar measures.

#### 2.2.2 VOSviewer

Relational networks were mapped using VOSviewer. We built co-authorship networks (countries, organizations, authors) and journal coupling networks (direct citation, bibliographic coupling, and co-citation). In these visualizations, the colors represent different clusters; the size of the nodes is proportional to the frequency of their occurrence in titles and abstracts; and the connections between the nodes represent the strength of collaboration or association. The minimum occurrence threshold was set to 5 for most analyses, and to 20 for the journal co-citation analysis to filter out fragmented data and maintain structural integrity. To prevent the actual results from being excessively dispersed due to inconsistent records in different databases, the data on countries and institutions were standardized before the network visualization. Detailed records are presented in Supplementary Tables 2, 3. We also visualized author keyword co-occurrences and density to identify thematic concentrations. Semantic redundancy was eliminated by systematically aggregating synonymous author keywords prior to network generation as described in Supplementary Table 4.

#### 2.2.3 CiteSpace

The spatio-temporal evolution of the field was obtained using CiteSpace. The parameters were set as follows: Timespan = 2005–2026, Slice Length = 1, g-index = 25, link retaining factor (LRF) = 2.5, maximum links per node (L/N) = 10, look back years (LBY) = 5, e = 1.0, link strength calculated by Cosine. We performed a journal dual-map overlay to map the flow of interdisciplinary knowledge and constructed reference citation networks. Similar to our VOSviewer protocol, we merged and pruned author keywords in CiteSpace. Prior to running the Log-Likelihood Ratio (LLR) clustering analysis for keywords, the alias and exclusion files were created to merge synonyms and hide irrelevant terms, respectively. This step prevents map contamination and increases the signal-to-noise ratio. The specific files are shown in Supplementary Tables 5, 6. The LLR algorithm was applied to cluster the author keywords and project them on a map of a chronological timeline. Moreover, burst detection was used to identify the 25 references and 25 keywords showing the strongest temporary increases in citation or occurrence activity. The software was calibrated to a standard g-index scaling factor. Finally, to assess the methodological reliability, sensitivity analyses were conducted on key bibliometric parameters. Burst strength and keyword prominence were interpreted as indicators of temporal scholarly attention and were not used as measures of methodological quality, causal evidence, therapeutic efficacy, or clinical utility.

#### 2.2.4 Other tools

Additional graphical engines were integrated to enhance the visual representation beyond the usual bibliometric visualizations. To make the clustering layout from VOSviewer clearer, it was changed with Pajek to organize it in columnar clusters. A Gephi-based chord diagram was developed to visualize the bilateral collaboration links among countries. The custom data visualization scripts were run on the PyCharm to produce a 3D ridgeline plot visualizing the volumetric publication changes of the core author keywords during last 21 years. To distinguish citation influence from evidentiary strength, we descriptively appraised the ten most locally cited documents according to study design, population, selenium exposure or intervention, cardiovascular outcomes, principal strengths, and major limitations. Because the studies were methodologically heterogeneous, no summary quality score was assigned; the appraisal is provided in Supplementary Table 7.

## 3 Results

### 3.1 Annual publication trends and global scientific production

**Figure 2:**
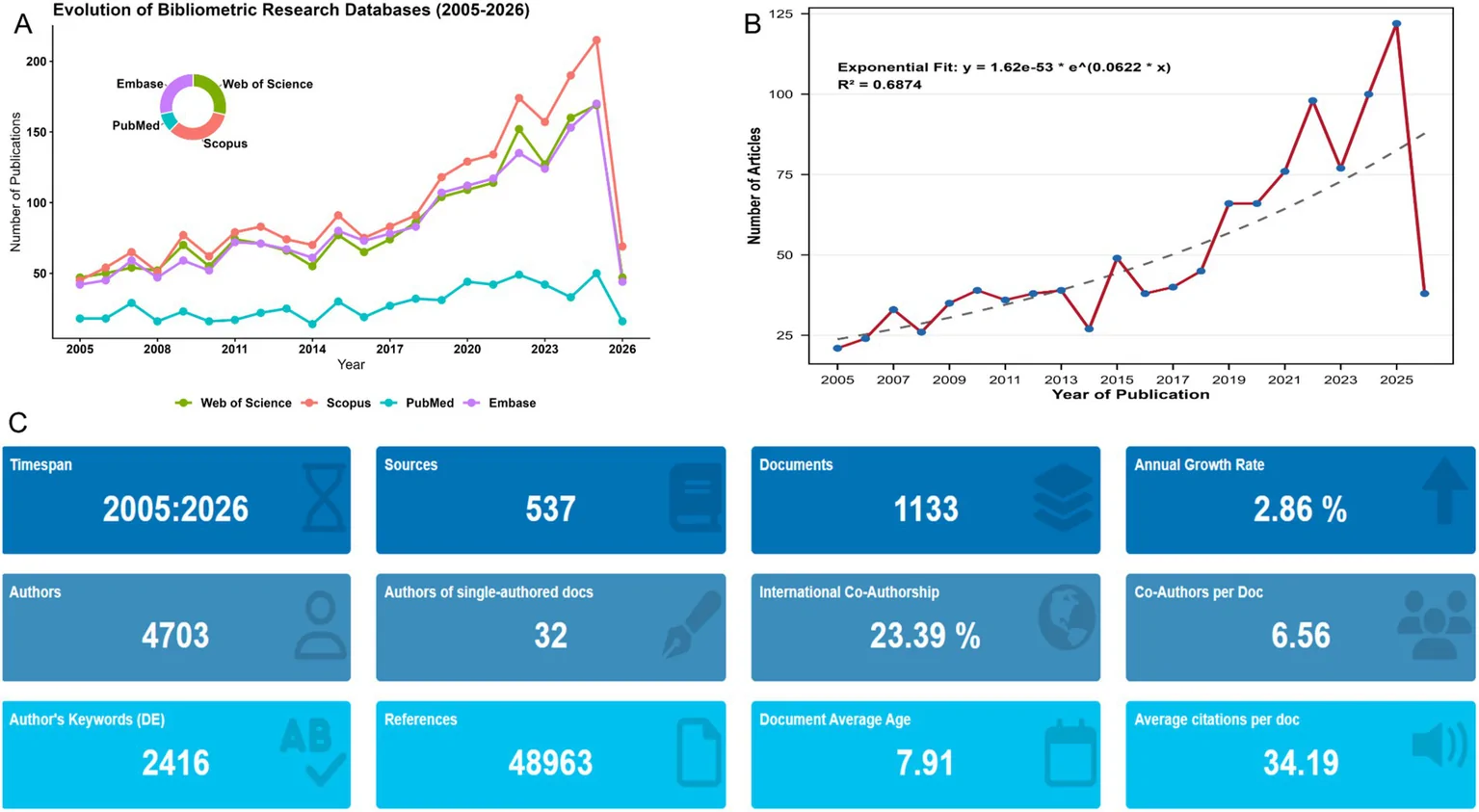
Annual publication trends and bibliometric overview of selenium-CVD research from 2005 to May 12, 2026. **(A)** Annual publication counts in Web of Science, Scopus, PubMed, and Embase; the 2026 values reflect partial-year indexing. **(B)** Exponential fit of annual publication output in the consolidated corpus, with the equation and coefficient of determination displayed in the panel. **(C)** Summary characteristics of the 1,133-document corpus.

The timeline of publications can be used to examine the evolution and development of selenium research in cardiovascular health. As depicted in Figure 2, the time axis from 2005 to 2026 is segmented into three phases.

In the first exploratory phase (2005–2013), the research output was limited and fluctuated across the four databases (Figure 2A). This is characteristic of the initial phase of knowledge accumulation. Then the field entered a steady growth phase (2014–2020), which was reflected by the continued upward rise in academic attention. Finally, in 2021, an expansion phase occurred. The number of publications boomed and the field became active.

This visual increase was statistically confirmed by performing a log-linear ordinary least squares (OLS) regression analysis on the combined publication data (Figure 2B). Since the 2026 data cannot represent the full-year publication volume, the equation fitting was only performed on the annual data from 2005 to 2025. The inherent inter-annual fluctuations are present, but the general trend is exponential (fitted equation: $y=20.39e^{0.0742t},t=\text{Year}-2004$ ) with high coefficient of determination $\;(R^{2}=0.856)$ and significant *p*-value ( p<0.001 ). This mathematical fit confirms the underlying upward trend of the field. Significantly, the raw temporal graph shows a decline in 2026. This is due to the fact that our retrieval results are exported on May 12, 2026 which cannot fully reflect the annual trend.

### 3.2 Bibliometric characteristics and general overview

This study offers a bibliometric overview of the research field from 2005 to 2026 (Figure 2C). During this period, a total of 1,133 valid documents were included, published in 537 different sources. The field showed a trend of steady development with an annual growth rate of 2.86 percent.

Authorship and collaboration patterns there were 4,703 scholars who contributed to the analyzed literature. The average number of authors per document was 6.56 and the international co-authorship rate was 23.39%. Also, 32 authors published single-authored documents. These figures show that team cooperation is the prevailing research paradigm in this field and that the share of cross-border academic exchange is relatively stable.

The corpus consisted of 2,416 author keywords (DE) and 48,963 references, which were indicative of knowledge accumulation and academic impact. The average number of citations per document was 34.19 and the average age of documents was 7.91 years. These measures demonstrate a consistent academic impact and ongoing citation activity for the research results in this field.

### 3.3 Global geographic contributions and collaborative networks

**Figure 3:**
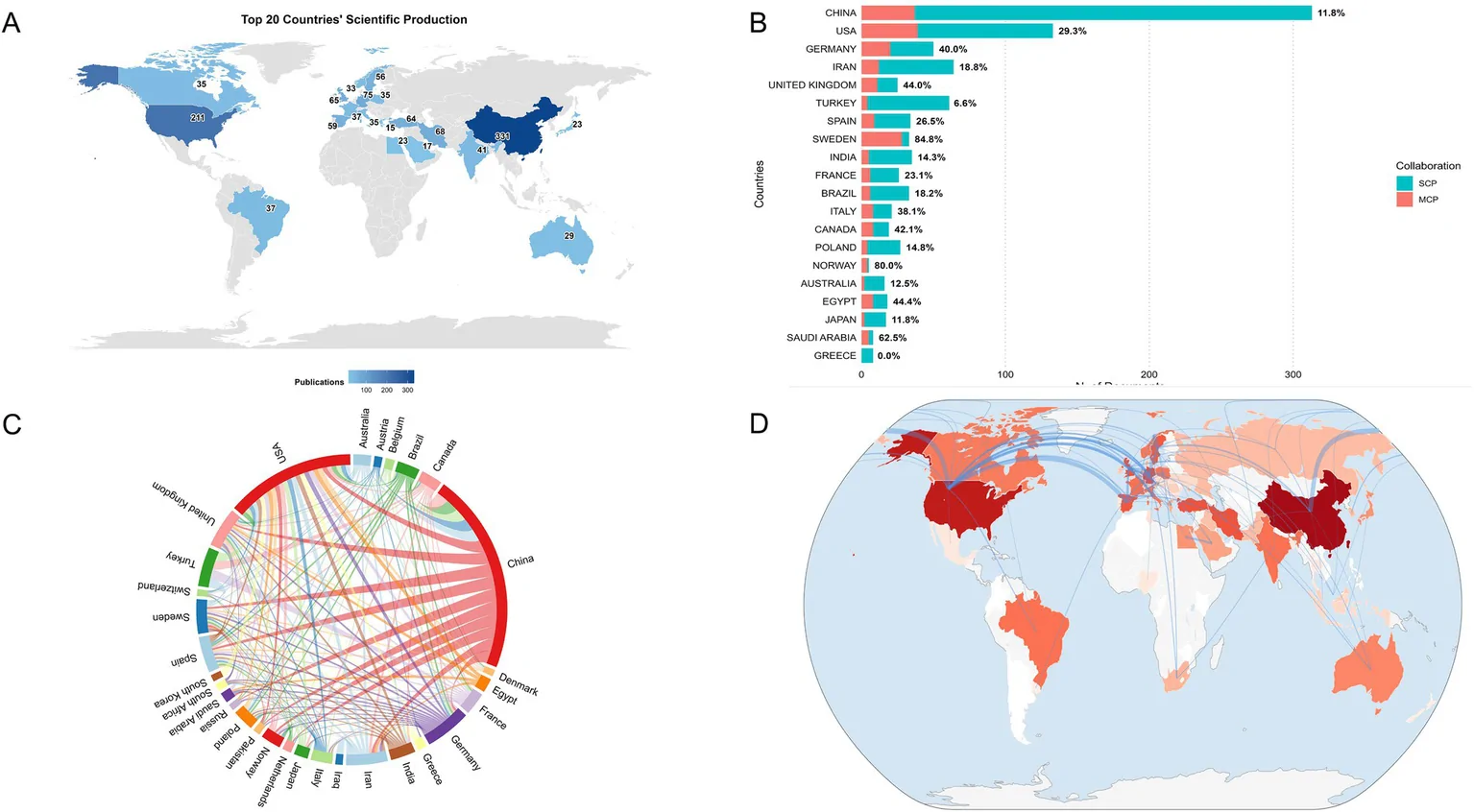
Global scientific production and international collaboration. **(A)** Geographic distribution of the 20 leading countries by publication output. **(B)** Single-country publications (SCP) and multiple-country publications (MCP), with MCP percentages shown. **(C)** Bilateral collaboration chord diagram. **(D)** Global cross-national collaboration map.

The geographic distribution of selenium–cardiovascular research was uneven (Figure 3A). China contributed the largest number of publications, with 331 records representing 29.21% of the included corpus, followed by the United States with 211 records (18.62%). Germany, Iran, the United Kingdom, Türkiye, Spain, Sweden, India, and France also ranked among the leading contributors. Table 1, Panel A summarizes publication volume, total citations, and citations per publication for the 20 most productive countries.

**Table 1:** The top 20 most productive countries, entities, authors among the included literature collection.

**Panel A: Countries**					**Panel B: Entities**						**Panel C: Authors**					
Rank	Country	TP	TC	TC/TP	Rank	Entity	Country	TP	TC	TC/TP	Rank	Author	Country	TP	TC	TC/TP
1	China	331	7,736	23.37	1	Charite Univ Med Berlin	Germany	30	775	25.83	1	Alehagen, Urban	Sweden	25	799	31.96
2	USA	211	12,032	57.02	2	Harvard Univ	USA	26	2,661	102.35	2	Schomburg, Lutz	Germany	23	448	19.48
3	Germany	75	4,210	56.13	3	Innlandet Hosp Trust	Norway	25	882	35.28	3	Alexander, Jan	Norway	19	490	25.79
4	Iran	68	953	14.01	4	Linkoping Univ	Sweden	25	848	33.92	4	Aaseth, Jan	Norway	16	659	41.19
5	United Kingdom	65	4,821	74.17	5	Johns Hopkins Univ	USA	24	2,270	94.58	5	Navas-Acien, Ana	USA	14	1,495	106.79
6	Turkey	64	1,129	17.64	6	Xi An Jiao Tong Univ	China	24	596	24.83	6	Larsson, Anders	Sweden	14	262	18.71
7	Spain	59	3,607	61.14	7	Huazhong Univ Sci & Technol	China	23	1,146	49.83	7	Stoppe, Christian	Germany	13	506	38.92
8	Sweden	56	1909	34.09	8	Norwegian Inst Publ Hlth	Norway	21	520	24.76	8	Guallar, Eliseo	USA	12	1737	144.75
9	India	41	848	20.68	9	Harbin Med Univ	China	19	387	20.37	9	Johansson, Peter	Sweden	11	497	45.18
10	France	37	1825	49.32	10	Inland Norway Univ Appl Sci	Norway	17	700	41.18	10	Stranges, Saverio	United Kingdom	9	1,017	113.00
11	Brazil	37	736	19.89	11	Uppsala Univ	Sweden	16	277	17.31	11	Muthukumaran, Azhaguchamy	India	8	292	36.50
12	Italy	35	1,667	47.63	12	Karolinska Inst	Sweden	13	587	45.15	12	Bomer, Nils	Netherlands	8	248	31.00
13	Canada	35	1,309	37.40	13	Shanghai Jiao Tong Univ	China	13	188	14.46	13	Van Der Meer, Peter	Netherlands	8	248	31.00
14	Poland	35	956	27.31	14	Jinan Univ	China	12	135	11.25	14	Loscalzo, Joseph	USA	7	1,603	229.00
15	Norway	33	1,254	38.00	15	Northeast Agr Univ	China	11	549	49.91	15	Turan, Belma	Turkey	7	237	33.86
16	Australia	29	1946	67.10	16	Chinese Acad Med Sci	China	11	401	36.45	16	Nogales, Fatima	Spain	7	95	13.57
17	Egypt	23	618	26.87	17	Ankara Univ	Turkey	11	392	35.64	17	He, Meian	China	6	303	50.50
18	Japan	23	430	18.70	18	Capital Med Univ	China	11	270	24.55	18	Beverborg, Niels Grote	Netherlands	6	230	38.33
19	Saudi Arabia	17	818	48.12	19	Brigham & Womens Hosp	USA	10	1733	173.30	19	Niu, Xiaolin	China	6	217	36.17
20	Greece	15	992	66.13	20	Univ Surrey	United Kingdom	10	869	86.90	20	Yang, Guang	China	6	143	23.83

TP = total publications. TC = total citations. TC/TP = average citations per publication. Records are primarily ranked by total publication output, followed by descending citation frequency for ties.

Patterns of domestic and international authorship differed across countries. China’s output was predominantly composed of single-country publications, whereas the United States, the United Kingdom, Germany, and several other European countries had higher proportions of multiple-country publications (Figure 3B). These findings describe differences in collaboration structure but do not establish why individual countries generated more publications. The present analysis did not include national selenium-status distributions, dietary intake, cardiovascular disease burden, research expenditure, or workforce capacity as explanatory covariates.

Publication volume and citation indicators also showed different geographic patterns. China had the highest number of publications, whereas the United States had the highest total citation count (TC = 12,032) and a higher mean citation rate (TC/TP = 57.02). The United Kingdom (TC/TP = 74.17) and Australia (TC/TP = 67.10) also showed high average citation rates. Such differences may reflect publication age, journal distribution, field composition, international visibility, and other factors that were not formally decomposed in the present analysis.

The bilateral collaboration chord diagram and global network map illustrate the principal cross-national connections (Figures 3C,D). The United States, the United Kingdom, Germany, and China occupied prominent positions in the international collaboration network, although the density and direction of their connections differed.

**Figure 4:**
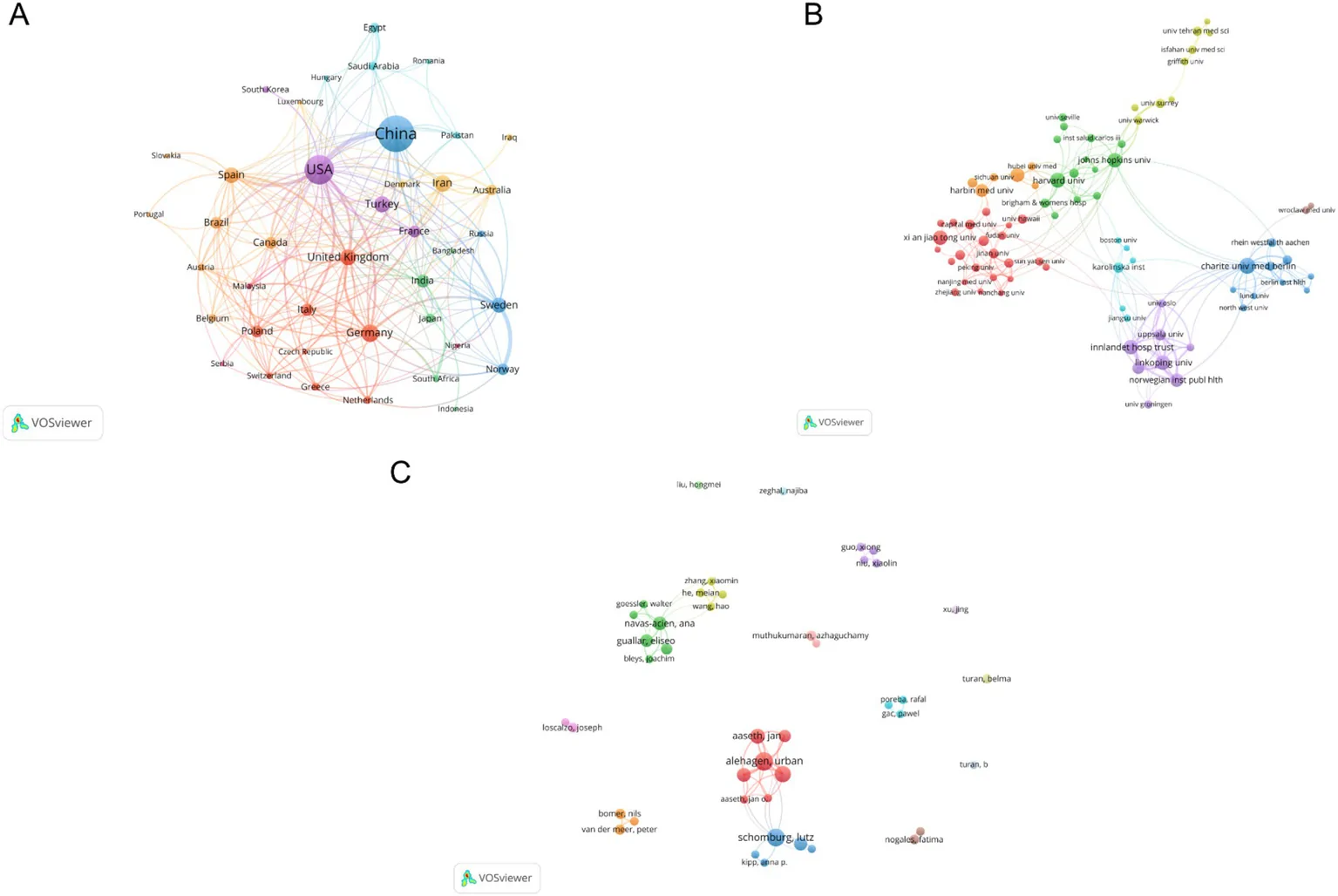
Co-authorship networks generated with VOSviewer. **(A)** Country-level network. **(B)** Institution-level network. **(C)** Author-level network. Node size reflects productivity, line thickness reflects collaboration strength, and colors indicate network clusters.

The country co-authorship network formed several clusters (Figure 4A). China was central to one cluster that included links with Sweden, Norway, Russia, and other countries. The United States was connected with partners including Türkiye, France, and South Korea, while the United Kingdom and Germany occupied central positions in a European collaboration cluster. Additional clusters included connections among Spain, Brazil, and Canada and a smaller network involving India and Japan. Cross-cluster links indicate that the field contains several regional collaboration structures rather than a single integrated global network.

### 3.4 Institutional hubs and academic capabilities

Also, the institutional landscape is characterized by a multi-centric geographical distribution. As shown in Table 1B, Charité - Universitätsmedizin Berlin (Germany, 30 publications) and Harvard University (USA, 26 publications) were the most productive hubs around the world. Nordic institutions, such as the Innlandet Hospital Trust (Norway) and Linköping University (Sweden), were also highly productive, with 25 articles each. The United States, Norway and Sweden each have three of the top 20 institutions by volume of publication, while eight are in China. The distribution shows the importance of regional research centers for the progress of cardiovascular-selenium research.

Importantly, American institutions dominated in scientific impact. Brigham & Women’s Hospital led the list with 173.30 citations per publication, followed by Harvard University (102.35), and Johns Hopkins University (94.58). The VOSviewer institutional network (Figure 4B) shows distinct clusters of collaboration: an integrated Western cluster (with Harvard, Charité, and Nordic universities at the centre) and an isolated, cohesive Chinese cluster (driven by Xi’an Jiaotong University and Huazhong University of Science and Technology), pointing to the significant contributions of Chinese and Western academic centers.

### 3.5 Core authors and intellectual landscapes

At the individual level, Table 1C measures the author’s impact by TP, TC, and TC/TP to identify the specific intellectual pioneers who are leading this field. The bibliometric analysis of selenium and cardiovascular research has identified 4,703 authors. Urban Alehagen (Sweden) leads the global production with 25 publications, followed closely by Lutz Schomburg (Germany, 23 publications) and Jan Alexander (Norway, 19 publications). But Joseph Loscalzo (USA) is the top ranked when evaluated by citation impact, with 229.00 citations per paper, followed by Eliseo Guallar (144.75) and Ana Navas-Acien (106.79).

The co-authorship network reveals 8 distinct author clusters. The top 20 authors by the number of publications include four authors in the red cluster and the other 14 authors distributed in the other seven clusters (Figure 4C), which reflects a decentralised architecture of collaboration. Such sparsity hints at an unexploited opportunity for cross-pollination between top research groups in this domain.

### 3.6 Leading journals and disciplinary focus

Table 2 lists the top 10 most productive journals in terms of total publications. Four of the top ten journals are based in Switzerland and three in the United States. The leading journal was Biological Trace Element Research, with 74 articles (6.53%), followed by the Journal of Trace Elements in Medicine and Biology (41) and Nutrients (37). The distribution shows that research continues to be concentrated in specialized publications in the fields of trace element and nutritional sciences. Among the top ten, Free Radical Biology and Medicine (IF = 8.2) had the highest academic impact with an average of 50.11 citations per paper. This supports oxidative stress and free radical mechanisms as recognized biological pathways linking selenium to cardiovascular health.

**Table 2:** The top 10 academic journals involving research related to Se and CVD.

Rank	Journal	Total Publications	Percent	Tota Citations	Average Citations	Country	IF Q2 2026
1	BIOLOGICAL TRACE ELEMENT RESEARCH	74	6.53%	1,445	19.53	USA	4.3
2	JOURNAL OF TRACE ELEMENTS IN MEDICINE AND BIOLOGY	41	3.62%	1,246	30.39	Germany	4.1
3	NUTRIENTS	37	3.27%	1,346	36.38	Switzerland	5.8
4	FRONTIERS IN NUTRITION	24	2.12%	565	23.54	Switzerland	5.5
5	ANTIOXIDANTS	18	1.59%	790	43.89	Switzerland	8.2
6	FREE RADICAL BIOLOGY AND MEDICINE	18	1.59%	902	50.11	USA	8.0
7	SCIENTIFIC REPORTS	18	1.59%	132	7.33	UK	4.9
8	INTERNATIONAL JOURNAL OF MOLECULAR SCIENCES	16	1.41%	543	33.94	Switzerland	5.6
9	PLOS ONE	14	1.24%	515	36.79	USA	2.8
10	ENVIRONMENTAL RESEARCH	13	1.15%	511	39.31	Netherlands	8.2

Q2 = second quarter (April–June) of 2026. Journals are primarily ranked by total publication output, followed by descending citation frequency for ties.

### 3.7 Inter-journal coupling dynamics and interdisciplinary knowledge flow

**Figure 5:**
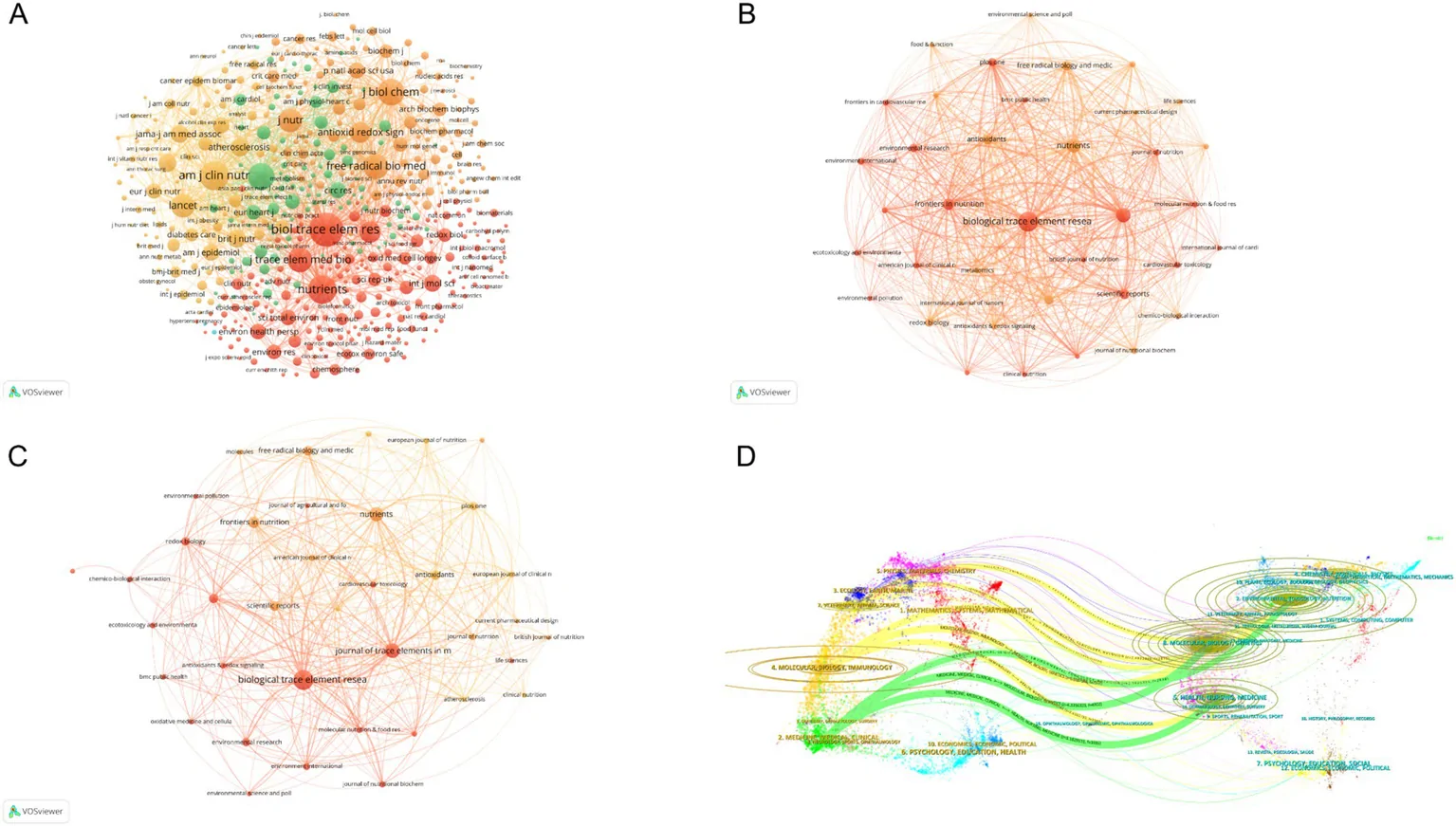
Journal and disciplinary knowledge landscape. **(A)** Journal co-citation network. **(B)** Journal bibliographic-coupling network. **(C)** Direct-citation network. **(D)** CiteSpace dual-map overlay showing citation flows from citing disciplines on the left to cited disciplines on the right.

To uncover the intellectual foundation of selenium and cardiovascular studies, we conducted a journal co-citation analysis (Figure 5A). We set a minimum citation threshold of 20 to filter out fragmented noise and reveal the foundational pillars of the field. The resulting high fidelity network clusters into distinct theoretical domains: clinical nutrition (e.g., Am J Clin Nutr), free radical biochemistry (e.g., Free Radical Bio Med) and core cardiovascular medicine (e.g., Atherosclerosis).

We visualized the bibliographic coupling (Figure 5B) and direct citation (Figure 5C) networks using the threshold 5, from historical roots to the current research front. Biological Trace Element Research and Nutrients forcefully occupy the central nodes in these dynamic landscapes. Their dense interconnectivity confirms that they are not only high-volume publishers, but the main conduits actively shaping the current academic conversation and methodological innovations.

We used CiteSpace to perform a dual-map overlay to track the macro trajectory of intellectual capital (Figure 5D). The map on the left shows the citing journals (the active research front) and the map on the right shows the cited journals (the foundational intellectual base). Colored trajectories are used to illustrate the directionality of knowledge flow. Our analysis suggests that selenium-cardiovascular research has operated as a complex, multi-pathway convergent and intersecting network.

First, the “2. Medicine, Medical, Clinical” cluster is one of the main frontier engines, and branches into three pathways. Its main trajectory settles on the base “8. Molecular, Biology, Genetics” (*z* = 4.336, *f* = 4,902). A clinical feedback loop feeds into “5. Health, Nursing, Medicine” (*z* = 3.183, *f* = 3,695) and a trajectory goes to “2. Environmental, Toxicology, Nutrition” (*z* = 2.393, *f* = 2,868).

“4. Molecular, Biology, Immunology” cluster is the second biggest engine of knowledge flow. It takes off on a dominant trajectory straight into “8. Molecular, Biology, Genetics” (*z* = 6.819, *f* = 7,502), the thickest and highest volume pathway on the whole map. In addition, the cluster extends to “5. Health, Nursing, Medicine” (*z* = 2.617, *f* = 3,102) and “2. Environmental, Toxicology, Nutrition” (*z* = 2.493, *f* = 2,973).

The cluster “7. Veterinary, Animal, Science” as a secondary knowledge source also steers its trajectories into “8. Molecular, Biology, Genetics” (*z* = 2.261, *f* = 2,730) and “2. Environmental, Toxicology, Nutrition” (*z* = 1.733, *f* = 2,179), especially. In terms of topology, clinically, molecular and veterinary sciences are deeply dependent on genomic and toxicological/nutritional data. In particular, the strong convergence towards the genetics base shows that current interdisciplinary research in this field is highly centered on the investigation of underlying mechanisms at the genetic and molecular levels.

### 3.8 Intellectual lineage and citation burst dynamics

**Figure 6:**
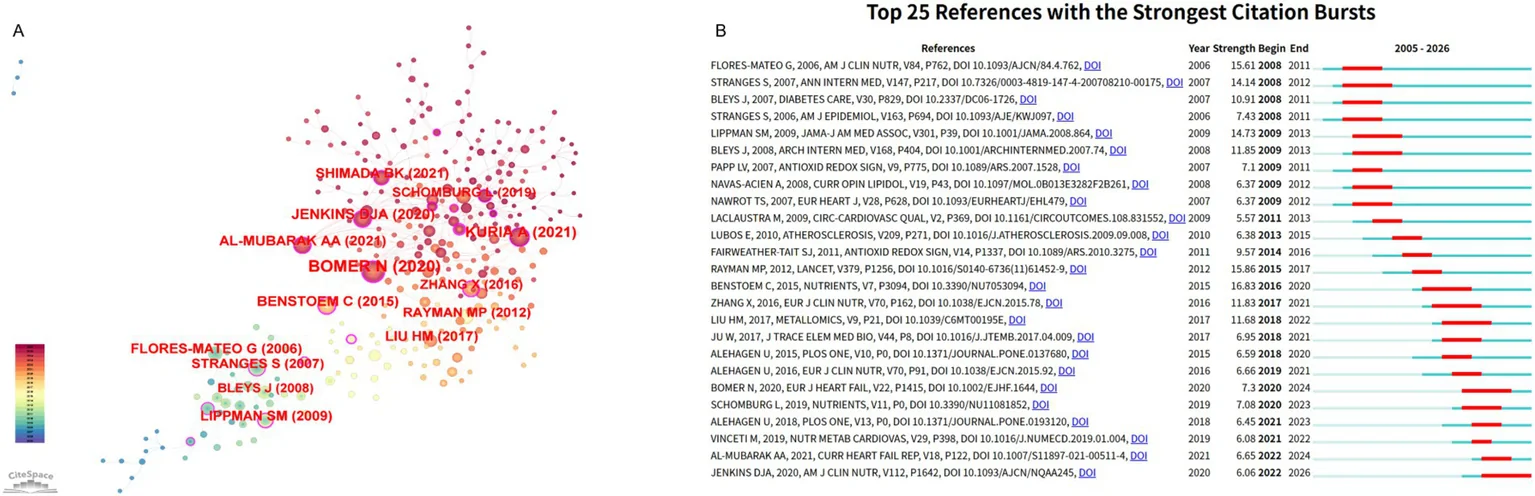
Reference co-citation network and citation-burst analysis. **(A)** Chronological co-citation network with representative influential references. **(B)** Top 25 references with the strongest citation bursts from 2005 to 2026; red segments indicate burst periods.

We constructed a reference co-citation network and applied citation-burst detection to describe the temporal structure of influential literature in the field (Figure 6). The co-citation network (Figure 6A) demonstrated the earlier concentration of the epidemiological, nutritional and supplementation-related references represented by publications such as Flores-Mateo, Stranges and Lippman. More recent nodes included studies by Bomer, Al-Mubarak, and Kuria, indicating increasing citation attention to heart failure, selenium status, prognosis, and phenotype-specific questions.

Citation-burst analysis identified the 25 references that experienced the most pronounced increases in citation activity during specific periods (Figure 6B). The strongest burst was for Benstoem et al. (31) (strength = 16.83; 2016–2020), then Rayman (7) (strength = 15.86) and Flores-Mateo et al. (22) (strength = 15.61). These burst periods reflect intervals during which the corresponding publications received rapidly increasing scholarly attention.

Several other recent references, including Jenkins et al. (25), whose citation burst persisted through the 2026 retrieval cutoff, and Al-Mubarak et al. (35) (strength = 6.65; 2022–2024), also showed sustained citation activity. Together, these trends indicate an increasing interest in selenium status, heart-failure phenotyping, prognosis and supplementation, and evidence a gradual shift towards more phenotype-specific and biomarker-oriented research themes.

### 3.9 Thematic architecture and spatiotemporal evolution of research hotspots

**Figure 7:**
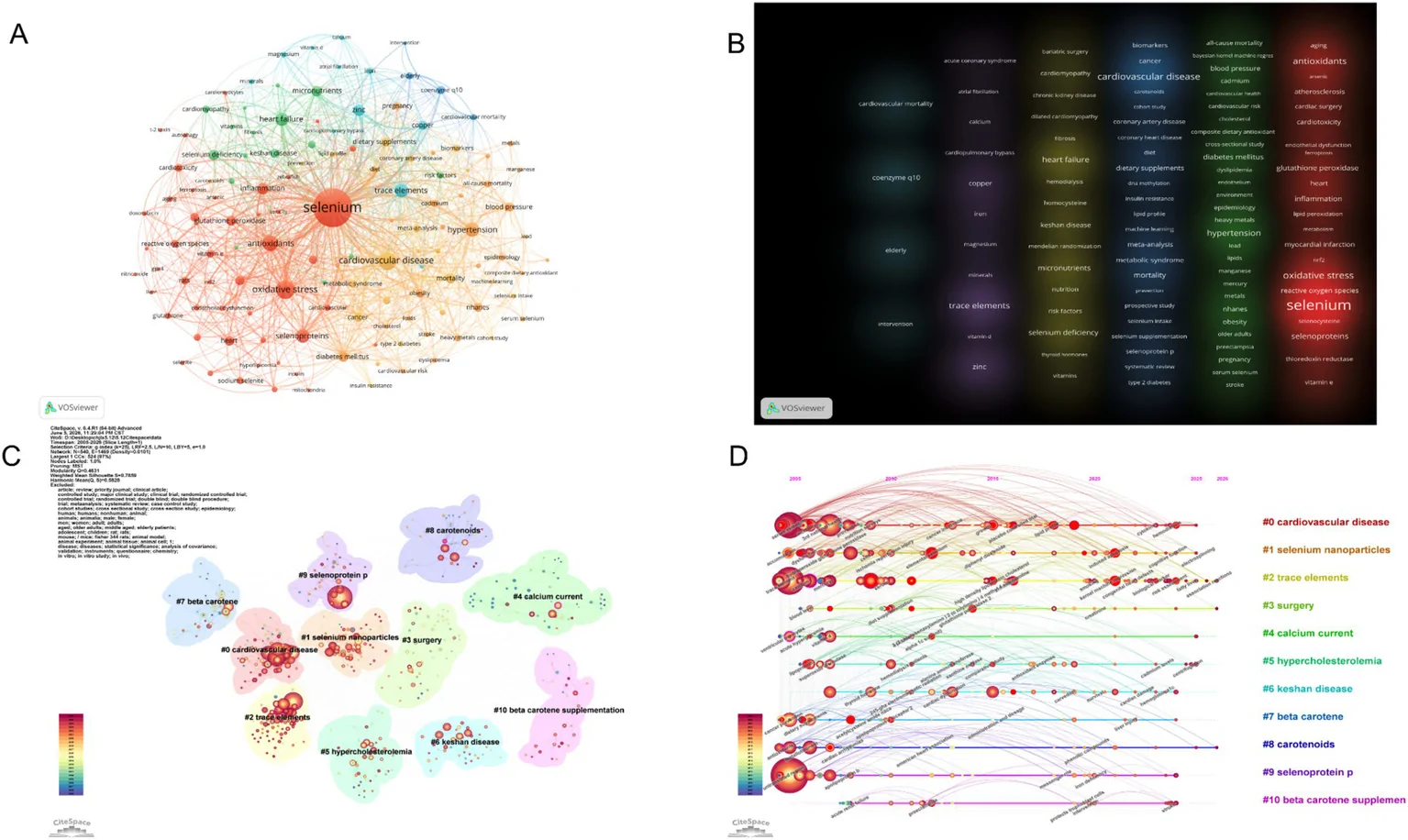
Keyword architecture and thematic evolution. **(A)** Author-keyword co-occurrence network. **(B)** Density visualization. **(C)** Log-likelihood ratio keyword clusters generated with CiteSpace. **(D)** Timeline visualization of the 11 keyword clusters.

To characterize the conceptual structure of the field, we generated an author-keyword co-occurrence network and density visualization using VOSviewer (Figures 7A,B). Synonymous terms were consolidated before network generation to reduce semantic redundancy. “Selenium” occupied the central position in the network and was frequently connected with “oxidative stress,” “antioxidants,” “inflammation,” and “glutathione peroxidase.” These associations indicate that redox-related concepts form a major thematic component of the literature and are frequently discussed alongside cardiovascular terms such as “heart failure” and “hypertension.”

Log-likelihood ratio clustering in CiteSpace identified 11 keyword clusters after the removal of non-specific terms (Figure 7C). The clusters covered broad cardiovascular topics, including #0 cardiovascular disease and #3 surgery, together with more specific mechanistic or biomarker-related topics such as #4 calcium current and #9 selenoprotein P. Cluster #1 captured the growing body of research concerning selenium nanoparticles.

Projection of the keyword clusters onto a chronological timeline (Figure 7D) illustrates how the thematic emphasis of the field changed over time. Earlier research was primarily concerned with endemic selenium deficiency, as reflected by the prominence of cluster #6, Keshan disease. More recent publications increasingly addressed selenium nanoparticles (#1) and metabolic topics related to hypercholesterolaemia (#5). Together, these temporal patterns indicate a shift in scholarly attention from deficiency-oriented public-health issues to molecular, metabolic, and material-science research themes.

### 3.10 Temporal evolution of thematic keywords and emerging frontiers

**Figure 8:**
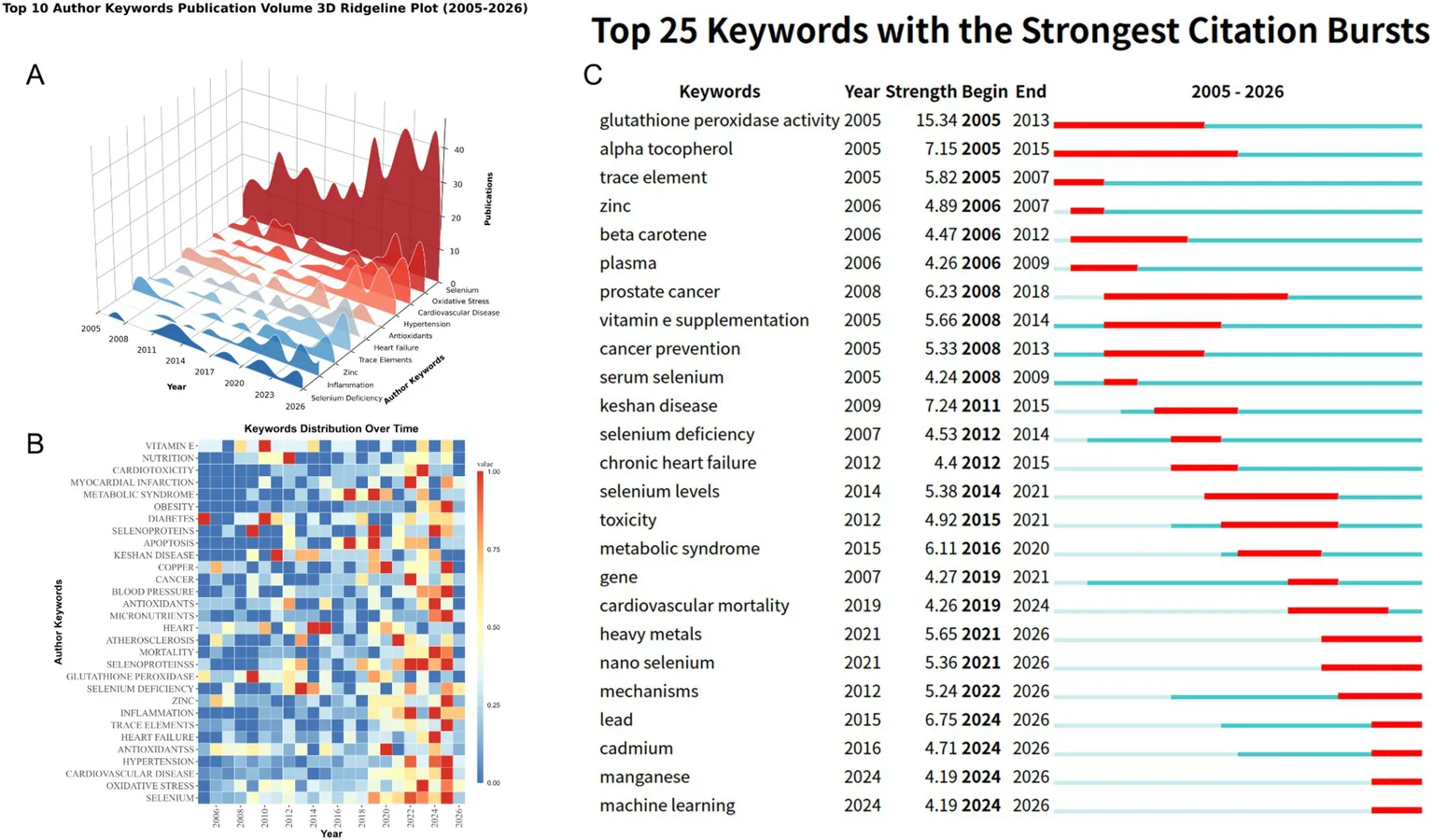
Temporal evolution of author keywords and emerging research fronts. **(A)** Three-dimensional ridgeline plot of annual publication volume for the 10 leading author keywords. **(B)** Heatmap of keyword distribution over time, normalized within each keyword. **(C)** Top 25 keywords with the strongest citation bursts from 2005 to 2026.

To examine the temporal trajectories of major research themes, we generated a three-dimensional ridgeline plot of annual keyword volume (Figure 8A) and a heatmap normalized to each keyword’s own publication history (Figure 8B). Synonymous variants were merged and non-specific methodological terms were removed before visualization.

The ridgeline plot shows that “selenium,” “oxidative stress,” and “antioxidants” maintained substantial publication volume across much of the study period. The normalized heatmap provides a complementary view by emphasizing when each term was most active relative to its own historical maximum. Earlier publications more frequently addressed Keshan disease, selenium deficiency, and nutrition, whereas publications from 2020 through the May 2026 retrieval cutoff increasingly included apoptosis, inflammation, metabolic syndrome, selenium nanoparticles, and computational methods.

Keyword-burst detection was used to identify terms that received rapidly increasing attention during specific periods (Figure 8C). The strongest and longest-lasting burst was observed for “glutathione peroxidase activity” (strength = 15.34; 2005–2013) reflecting the early emphasis on selenium-related antioxidant biology. More recent bursts extending to the 2026 retrieval cutoff involved environmental co-exposure terms including “heavy metals,” “lead,” “cadmium,” and “manganese,” together with “nano selenium” (strength = 5.36) and “machine learning.” These patterns indicate growing publication activity in environmental toxicology selenium-nanoparticle research and computational approaches within the selenium–cardiovascular literature.

### 3.11 Sensitivity Analysis

To verify the stability of our findings, sensitivity analyses were performed using varying parameter thresholds. The results demonstrated that the core clustering structures and the detection of major citation bursts remained highly robust. Detailed visual networks and evaluation metrics are provided in Supplementary Figures (Sensitivity Analyses).

## 4 Discussion

### 4.1 Principal findings and interpretive framework

The study mapped 1,133 publications retrieved from four databases and found that the research on selenium and CVDs significantly increased after 2015. The intellectual structure stayed grounded in selenium deficiency, antioxidant enzymes, oxidative stress, and nutritional epidemiology, but recent themes have increasingly focused on heart failure, SELENOP, GPX4, ferroptosis, nano-selenium, environmental co-exposures, and machine learning. The geographic and collaboration analyses also showed an uneven distribution of publication volume, citation influence, and international connectivity.

The main interpretation is biological coherence but translational fragmentation in the field. Mechanistic studies suggest plausible roles for selenoproteins in redox regulation and lipid-peroxide control; observational studies often associate low or atypical selenium status with adverse cardiovascular phenotypes; randomized evidence does not support a broadly applicable preventive effect. These layers of evidence address different questions. Consistency should not be sought by prioritizing one design, but by determining the populations, biomarkers, doses, formulations and endpoints to which each finding applies.

### 4.2 From deficiency cardiomyopathy to functional and precision-oriented research

Temporal patterns indicate a shift from deficiency-driven research to functional biomarker and phenotype-driven research. The early prominence of Keshan disease, glutathione peroxidase activity and antioxidant defense reflected a clinically recognizable deficiency model. The later emphasis on mortality, heart failure, SELENOP, GPX4 and ferroptosis illustrates that the field is increasingly asking how selenium-dependent functions differ between disease states and if such differences are of prognostic or therapeutic relevance.

The structure of the citation supports the same interpretation. Broad selenium biology, redox, speciation and human-health reviews dominated the most globally cited documents (Table 3). The most locally cited documents in the selenium-CVD corpus were focused on coronary disease, cardiovascular mortality, heart failure and supplementation (Table 4). Thus global citation influence reflects the general biochemical basis of the field, while local citation influence more explicitly identifies the clinical controversies structuring this literature. The accompanying design-level appraisal showed that these locally influential publications included heterogeneous observational, interventional, and evidence-synthesis designs with different risks of confounding, bias, and limited generalizability; their citation prominence therefore did not represent a uniform level of methodological strength (Supplementary Table 7).

**Table 3:** The 10 most globally cited documents among the included literature collection.

First author	Country	Title	Document type	Journal	Year	Global citations
Fairweather-Tait S	United Kingdom	Selenium in Human Health and Disease	Review	Antioxidants and Redox Signaling	2011	1,063
Lubos E	USA	Glutathione Peroxidase-1 in Health and Disease: From Molecular Mechanisms to Therapeutic Opportunities	Review	Antioxidants and Redox Signaling	2011	1,044
Navarro-Alarcon M	Spain	Selenium in Food and the Human Body: A Review	Review	Science of the Total Environment	2008	727
Weekley C	Australia	Which Form Is That? The Importance of Selenium Speciation and Metabolism in the Prevention and Treatment of Disease	Review	Chemical Society Reviews	2013	562
Fernández-Martínez	France	Selenium Environmental Cycling and Bioavailability: A Structural Chemist Point of View	Review	Reviews in Environmental Science and Biotechnology	2009	447
Sies H	Germany	Nutritional, Dietary and Postprandial Oxidative Stress	Article	Journal of Nutrition	2005	439
Steinbrenner H	Germany	Protection Against Reactive Oxygen Species by Selenoproteins	Review	Biochimica et Biophysica Acta-General Subjects	2009	404
Battin E	USA	Antioxidant Activity of Sulfur and Selenium: A Review of Reactive Oxygen Species Scavenging, Glutathione Peroxidase, and Metal-Binding Antioxidant Mechanisms	Review	Cell Biochemistry and Biophysics	2009	396
Beckett G	France	Selenium and Endocrine Systems	Review	Journal of Endocrinology	2005	383
Zoidis E	Greece	Selenium-Dependent Antioxidant Enzymes: Actions and Properties of Selenoproteins	Review	Antioxidants	2018	364

Ranked in descending order by global citation count.

**Table 4:** The 10 most locally cited documents among the included literature collection.

First author	Country	Title	Document type	Journal	Year	Local citations
Flores-Mateo G	USA	Selenium and Coronary Heart Disease: A Meta-Analysis	Article	American Journal of Clinical Nutrition	2006	121
Bleys J	USA	Serum Selenium Levels and All-Cause, Cancer, and Cardiovascular Mortality Among US Adults	Article	Archives of Internal Medicine	2008	83
Benstoem C	Germany	Selenium and Its Supplementation in Cardiovascular Disease-What Do We Know?	Review	Nutrients	2015	76
Bomer N	Netherlands	Selenium and Outcome in Heart Failure	Article	European Journal of Heart Failure	2020	59
Zhang X	USA	Selenium Status and Cardiovascular Diseases: Meta-Analysis of Prospective Observational Studies and Randomized Controlled Trials	Article	European Journal of Clinical Nutrition	2016	55
Stranges S	USA	Effects of Selenium Supplementation on Cardiovascular Disease Incidence and Mortality: Secondary Analyses in a Randomized Clinical Trial	Article	American Journal of Epidemiology	2006	51
Laclaustra M	USA	Serum Selenium Concentrations and Hypertension in the US Population	Article	Circulation-Cardiovascular Quality and Outcomes	2009	47
Alehagen U	Sweden	Cardiovascular Mortality and N-Terminal-proBNP Reduced After Combined Selenium and Coenzyme Q10 Supplementation: A 5-Year Prospective Randomized Double-Blind Placebo-Controlled Trial Among Elderly Swedish Citizens	Article	International Journal of Cardiology	2013	46
Kuria A	Sweden	Selenium Status in the Body and Cardiovascular Disease: A Systematic Review and Meta-Analysis	Review	Critical Reviews in Food Science and Nutrition	2021	40
Bleys J	USA	Serum Selenium and Peripheral Arterial Disease: Results from the National Health and Nutrition Examination Survey, 2003–2004	Article	American Journal of Epidemiology	2009	36

Ranked in descending order by local citation count.

This transition raises the evidentiary bar. Correcting an obvious deficit sometimes prevents a severe deficiency syndrome. In contrast, small differences in circulating selenium in contemporary populations may be related to diet, inflammation, kidney function, disease severity, changes in protein synthesis or redistribution. Thus the change in the bibliometric landscape should be seen as a sign of maturation of the research question, not as an indication that precise selenium intervention is ready for routine application.

### 4.3 Mechanistic plausibility does not establish clinical efficacy

**Figure 9:**
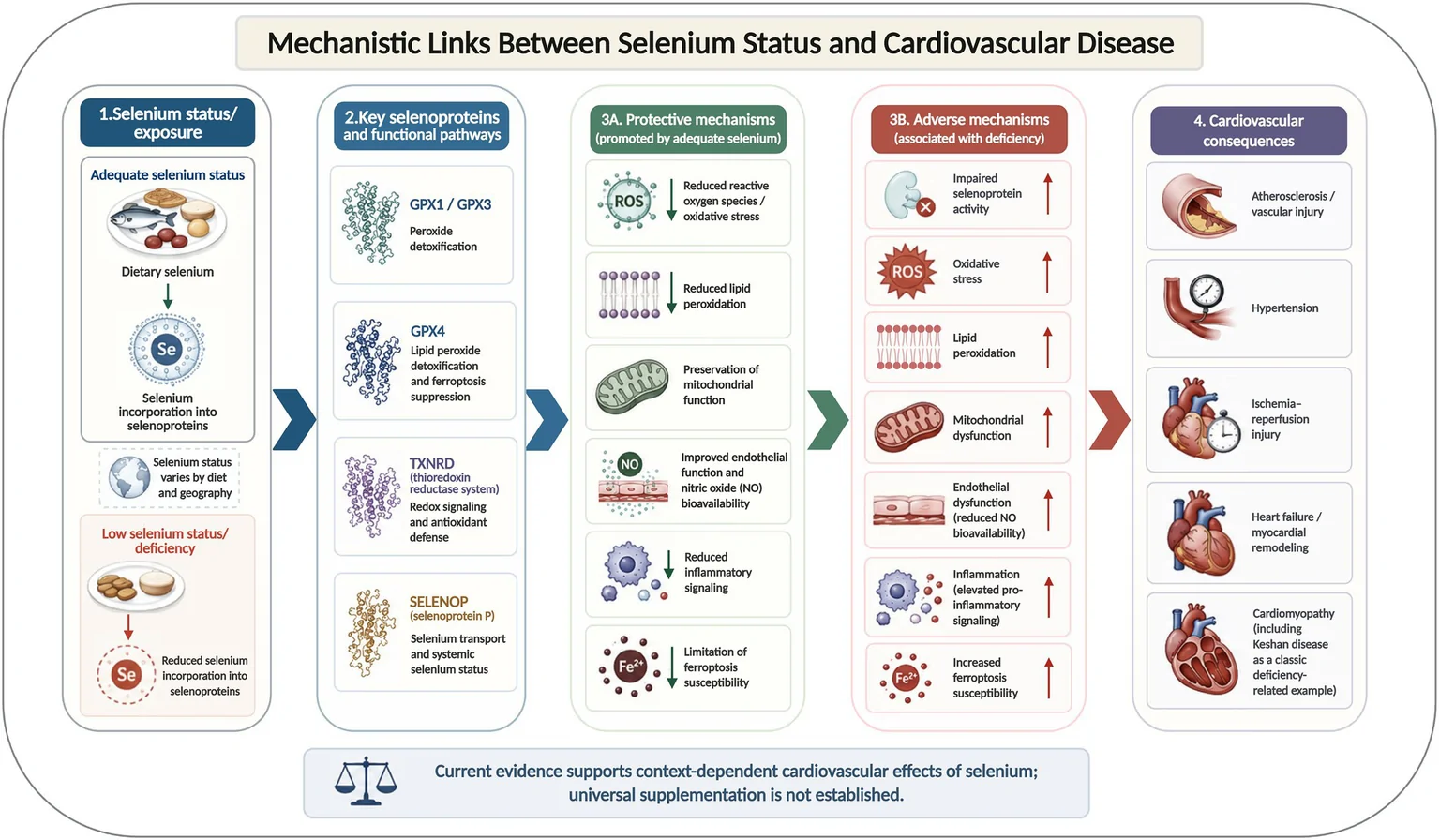
Mechanistic links between selenium status and cardiovascular disease. The framework distinguishes selenium exposure, key selenoproteins and functional pathways, protective processes associated with adequate selenium status, adverse processes associated with deficiency, and potential cardiovascular consequences. The framework summarizes biological plausibility and does not establish that supplementation prevents cardiovascular events. Heuristic mechanistic model of potential links between selenium status and cardiovascular disease. The model distinguishes selenium exposure, selenoprotein-related functions, candidate biological pathways, and potential cardiovascular consequences. It is intended to organize biological hypotheses and does not represent a validated causal pathway, treatment algorithm, or evidence that selenium supplementation prevents cardiovascular events.

Oxidative stress, redox signaling, endothelial activation, and inflammation contribute to atherosclerosis and myocardial injury (51–53). Selenium-dependent enzymes provide a plausible connection because glutathione peroxidases and thioredoxin reductases participate in peroxide reduction and redox regulation (5, 8, 10, 11, 31–33). However, reactive species have physiological signaling roles and extra selenium intake does not in itself correct a disease-specific defect when selenium supply is already adequate for selenoprotein synthesis. The mechanistic framework of Figure 9 should therefore be taken as a map of potential pathways rather than evidence of efficacy of supplementation.

The emergence of GPX4 and ferroptosis illustrates this distinction. GPX4 limits phospholipid hydroperoxide accumulation (54), while ferroptosis is an iron-dependent form of regulated cell death driven by lipid-peroxide failure (55, 56). Cardiovascular reviews have proposed roles for ferroptosis in atherosclerosis, ischemia–reperfusion injury, cardiomyopathy, and heart failure (57, 58). These findings support pathway-specific research, but do not prove that oral selenium prevents ferroptosis-mediated cardiovascular events in humans. Translation requires evidence that the pathway is selenium limited in the target population, that an intervention alters a validated functional marker, and that this alteration improves a pre-specified clinical endpoint.

### 4.4 Baseline status and functional biomarkers as potential effect modifiers

There is biological plausibility for effect modification by baseline selenium status. Participants with higher selenium status may have limited capacity to respond further functionally to supplementation, whereas those with lower baseline status may have greater capacity for a measurable increase in selenoprotein expression or activity. This interpretation is consistent with SELENOP being a transport protein and systemic status biomarker (9, 59), with methodological and intervention studies showing that total selenium and functional indicators can respond differently (60–62), and with the absence of consistent cardiovascular benefit in broadly supplemented populations (25–27).

Total serum or plasma selenium is useful for population comparisons, but it does not capture selenium species, tissue delivery, or functional incorporation (60, 61). SELENOP reflects hepatic synthesis and systemic transport (9, 59); extracellular GPx activity represents one antioxidant function (60–62); and GPX4 is a cellular enzyme involved in phospholipid-hydroperoxide control rather than a routine circulating status biomarker (54). Future studies should therefore state whether the intended construct is recent intake, long-term exposure, transport capacity, enzymatic function, or tissue-specific biology, and should avoid treating these measures as interchangeable.

Heart failure is a particularly informative setting. Low selenium has been associated with poorer exercise capacity and outcomes in heart failure (36), and reviews have outlined plausible myocardial mechanisms (35). Prospective studies have associated low SELENOP with incident heart failure and mortality (37–39), while transcriptomic work has linked serum selenium status with immunoregulatory processes (34). Additional hospitalized-cohort analyses associated low SELENOP with anemia and cognitive impairment (63, 64). These results may identify a vulnerable phenotype, but reverse causation remains possible because advanced disease can alter appetite, absorption, inflammation, hepatic protein synthesis, renal handling, and volume status.

At present, there are no universally accepted cardiovascular cut-points for SELENOP or extracellular GPx/GPX3 activity. Reported concentrations and activities are dependent on platform, assay calibration, reference population, pre-analytical conditions, inflammatory state, hepatic synthesis, renal function and acute illness. Thus, values obtained with different analytical approaches are not directly interchangeable (65–68).

Assay-specific thresholds, however, may be considered as exploratory enrichment criteria in early-phase trials. In the immunoluminometric assay of Hollenbach et al., the median SELENOP concentration in healthy individuals was 3.04 mg/L with an interquartile range of 2.6–3.4 mg/L (65). Thus, a lower-quartile criterion of less than 2.6 mg/L could be used as an example in studies using the same assay method.

In the ESTHER cohort of older German adults, mortality risk was elevated at SELENOP concentrations below about 4.1 mg/L, close to the lower-tertile limit of 4.2 mg/L (39). This value may offer an independent risk-derived candidate threshold for studies using the same calibrated monoclonal ELISA in similar populations. The estimates were obtained from different assays and populations and should not be interpreted as interchangeable cardiovascular thresholds (39, 66, 68).

For plasma extracellular GPx activity, a universal absolute threshold is even less defensible because enzyme assays differ in substrate, reaction conditions, calibration, and reporting units. A reference study using a modified Paglia–Valentine method reported a plasma GPx activity interval of 196–477 U/L in healthy adults (69). The lower boundary of 196 U/L may be considered only as a method-specific illustrative criterion when the same analytical procedure is used. More generally, low extracellular GPx activity should be defined relative to a validated laboratory-specific reference distribution and confirmed in a second sample.

On-treatment target engagement should be prospectively defined as a change greater than the analytical imprecision of the assay and, where validated analyte-specific biological-variation data are available, the corresponding reference change value, together with movement toward the assay-specific reference range or evidence of a biomarker-response plateau (70, 71). Biological-variation data are available for total serum selenium in some populations. Reference change values for SELENOP and extracellular GPx/GPX3 are limited.

Serial assessment should include total selenium, SELENOP, and extracellular GPx activity, as well as inflammatory markers, serum albumin, hepatic function, and renal function (39, 65, 67). This combined approach may help distinguish selenium-responsive insufficiency from low biomarker concentrations related primarily to disease severity, inflammation, impaired protein synthesis, or altered distribution.

Future trials should prespecify treatment-by-baseline-status interaction analyses and examine selenium biomarkers as continuous variables, using nonlinear models where appropriate. Categorical thresholds may support trial enrichment, but they should not replace continuous analyses or prospective validation. Accordingly, the proposed ranges are most appropriately evaluated as assay-specific enrichment and response variables in prospective trials.

### 4.5 Cardiovascular endpoints and exposure metrics should remain distinct

Coronary heart disease, stroke, hypertension, heart failure, cardiomyopathy, perioperative injury, and cardiovascular mortality are not synonymous outcomes. Their dominant mechanisms, latency, competing risks and susceptibility to nutritional exposures are different. This also applies to dietary intake, serum selenium, SELENOP, GPx activity and supplementation. Pooling heterogeneous outcomes or exposures may increase statistical power but may obscure clinically important differences.

The existing literature demonstrates this heterogeneity. Earlier syntheses evaluated coronary disease or broad cardiovascular outcomes (22–24), whereas more recent work has focused on cause-specific mortality (21), SELENOP and heart failure (34, 37–39), selenium distribution across functional proteins (40), ischemic stroke (42–44), and dietary selenium in composite CVD or chronic-disease outcomes (41, 45). In adults with type 2 diabetes or chronic kidney disease, higher serum selenium was associated with lower mortality in observational analyses (72, 73). A hypertensive-population analysis reported a U-shaped association (74), and a nested case–control study within PREDIMED did not support a simple linear protective relation for serum selenium and incident CVD (75). A recent meta-analysis of serum selenium and lipid profiles further showed that biomarker-outcome relations differ across populations and analytic designs (76). These findings support endpoint-specific and biomarker-specific estimates rather than a universal selenium effect.

### 4.6 Supplementation evidence and the limits of broad prevention

Randomized evidence is the appropriate test of supplementation, but interpretation must be population and intervention specific. Secondary analyses of the Nutritional Prevention of Cancer trial did not show an overall reduction in CVD incidence or mortality with selenium supplementation (27). SELECT was designed primarily for cancer prevention and enrolled a largely selenium-replete population; it did not establish cardiovascular benefit and should not be treated as a dedicated cardiovascular efficacy trial (77). A controlled study in healthy North American men found no improvement in vascular responsiveness (78). Trial-based systematic reviews consequently do not support routine selenium supplementation for primary cardiovascular prevention (25, 26).

The Swedish trial of combined selenium and coenzyme Q10 reported lower cardiovascular mortality during intervention and extended follow-up (79, 80). Secondary analyses suggested stronger effects among participants with lower baseline selenium and reported associations with telomere attrition and coagulation-related biomarkers (81–83). Clinically interesting but cannot tease out selenium as both nutrients were given together. The population was older, geographically specific and relatively selenium deficient, limiting extrapolation to younger or selenium-replete groups. The findings support replicating a status-stratified combined intervention rather than universal use of selenium.

Perioperative studies are a different clinical scenario. A 2024 meta-analysis of randomized trials of cardiac surgery assessed short-term clinical and biochemical outcomes but did not show a consistent mortality benefit (84). Critical illness and cardiopulmonary bypass can change trace-element concentrations by inflammation, redistribution, dilution and oxidative stress. Results from this setting may not be applicable to long-term primary prevention. Future trials should report baseline status, selenium formulation, dose, adherence, achieved biomarker change, co-interventions, adverse events and clinically meaningful endpoints.

### 4.7 Nonlinear dose–response, safety, and metabolic trade-offs

The nutritional interpretation of selenium is non-linear by nature. Beyond physiological requirements, little additional functional gain is available from exposure, whereas inadequate selenium availability may limit selenoprotein synthesis and function (3, 7). Excess selenium exposure can lead to toxicity (7, 85), and observational data have raised concerns regarding adverse metabolic associations in some selenium-replete populations (30). Additional evidence from meta-analyses of mortality, inflammatory markers, blood pressure and lipid profiles also suggest selenium associations and supplementation responses are dependent on baseline status, exposure level, population and outcome (21, 28, 29).

Quantitative analyses available demonstrate this non-linearity but do not point to a universal cardiovascular selenium target. In a meta-analysis of prospective studies, a nonlinear association between blood selenium concentrations and risk of cardiovascular disease was reported for the range of approximately 30–165 μg/L, while an inverse association was observed for the narrower range of approximately 55–145 μg/L. However, the analysis combined heterogeneous populations, cardiovascular endpoints and selenium biomarkers and the reported range should not be considered as a clinical reference interval or supplementation target. The corresponding analysis of randomized trials did not demonstrate a significant reduction in cardiovascular disease with selenium supplementation (24).

More recent population studies have detected different inflection points depending on the exposure metric and outcome. Cross-sectional analysis of NHANES data suggested that dietary selenium intake was nonlinearly associated with prevalent cardiovascular disease, and the estimated inflection point was 135.28 μg/day. This estimate does not demonstrate temporality and cannot be directly converted into a circulating selenium target, as dietary intake and prevalent cardiovascular disease were assessed using a cross-sectional design (41). In adults with hypertension, serum selenium showed U-shaped associations with all-cause and cardiovascular mortality, with estimated risk nadirs at approximately 136 and 130 μg/L, respectively. These estimates were derived from a specific NHANES subgroup and should be regarded as population- and outcome-specific rather than universal thresholds (74).

Intervention-based dose–response evidence also indicates that metabolic responses may differ according to administered dose, achieved selenium concentration, baseline status, and selenium formulation. A dose–response meta-analysis of experimental human studies found that selenium administration at or above approximately 200 μg/day was associated with lower HDL and LDL cholesterol and higher triglyceride concentrations. When achieved blood selenium concentrations exceeded approximately 150 μg/L, triglyceride and LDL cholesterol concentrations tended to increase, whereas HDL cholesterol decreased. Associations were generally stronger for inorganic selenium formulations than for organic selenium formulations (86). These mixed lipid responses support the value of assessing individual metabolic endpoints rather than classifying all biomarker changes as uniformly beneficial or adverse.

**Figure 10:**
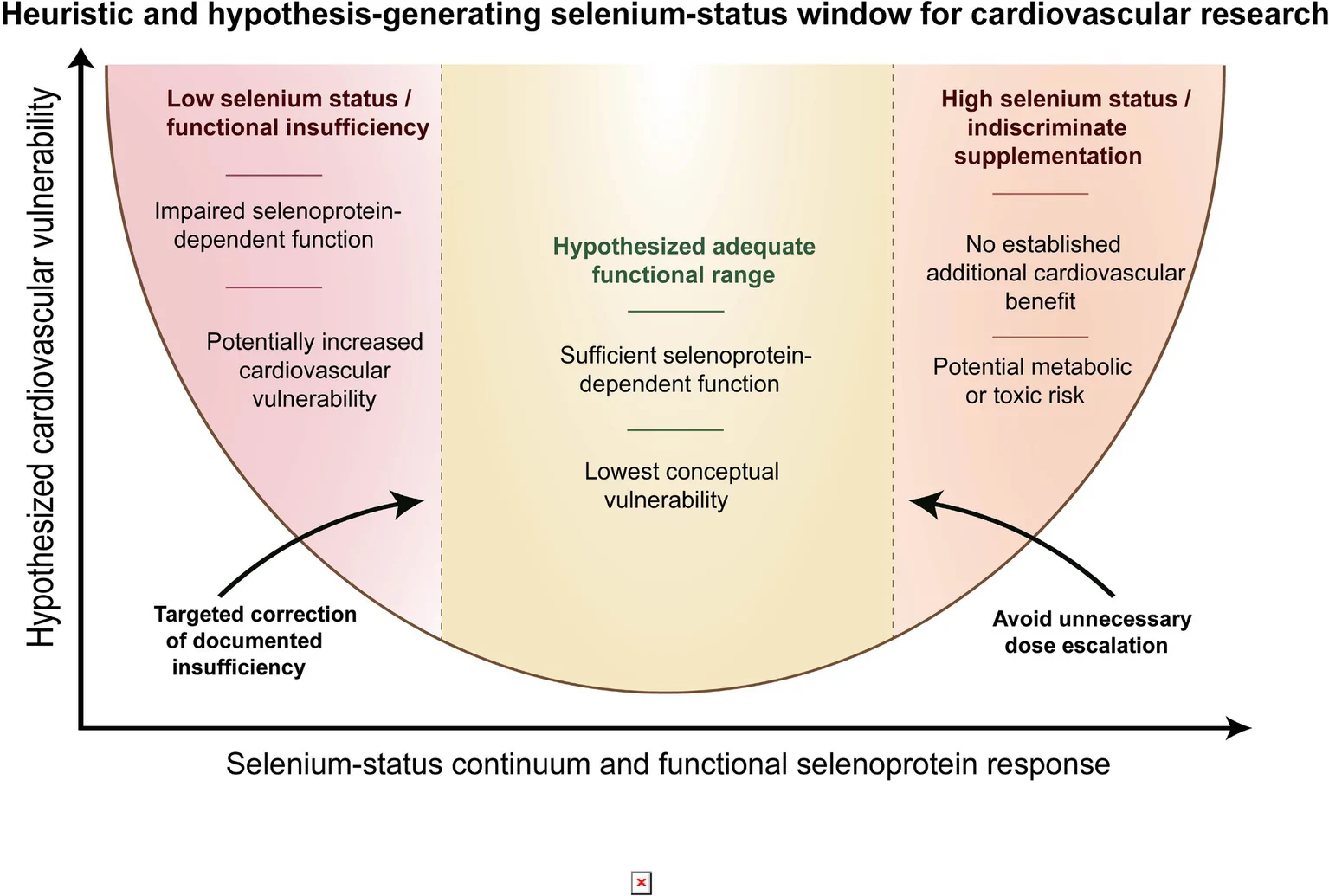
Conceptual selenium-status window for cardiovascular prevention. Low selenium status or functional insufficiency may be associated with greater vulnerability; an adequate functional range is hypothesized to support selenoprotein-dependent physiology; and high selenium status or indiscriminate supplementation is not presumed to provide additional benefit and may entail metabolic or toxic risk. This is a conceptual framework, not a quantitative dose–response estimate or clinical threshold. Heuristic and hypothesis-generating selenium-status window for cardiovascular research. Low selenium status or functional insufficiency may be associated with increased vulnerability, whereas an adequate functional range is hypothesized to support selenoprotein-dependent physiology. Higher status or indiscriminate supplementation is not assumed to provide additional cardiovascular benefit and may entail metabolic or toxic risk. The boundaries are intentionally non-numerical because estimates differ by exposure metric, assay, population, disease phenotype, and endpoint. The figure is not a quantitative dose–response curve, clinical reference interval, supplementation target, or treatment recommendation.

Taken together, these findings support a testable selenium-status window rather than a single universal target (Figure 10). At the lower end, inadequate intake, impaired selenium transport, or insufficient selenoprotein function may be associated with increased vulnerability. The middle range represents hypothesized functional sufficiency, beyond which additional exposure may provide little cardiovascular benefit (116). At the upper end, unnecessary supplementation or excessive exposure may increase metabolic or toxic risk. The locations of these transition zones are likely to vary according to the exposure measure, analytical assay, geographic and dietary context, age, cardiovascular phenotype, comorbidity, selenium formulation, and outcome. Dietary selenium intake, total serum or plasma selenium, SELENOP, extracellular GPx activity, and supplemental dose are related but non-equivalent measures and should not be combined into a single numerical cut-point. Prospective studies are needed to establish biomarker- and population-specific boundaries.

### 4.8 Interpreting emerging hotspots: environmental co-exposures, nano-selenium, and machine learning

Recent keyword activity highlights three increasingly interconnected themes: environmental metal co-exposures, nano-selenium formulations, and machine-learning applications. Together, these themes broaden the selenium–cardiovascular literature from conventional single-exposure analyses toward mixture-based exposure assessment, formulation-specific biological investigation, and multivariable modelling of cardiovascular phenotypes.

Environmental co-exposure research may better approximate real-world conditions, in which selenium occurs alongside both essential elements and toxic metals. Recent studies have also assessed multiple metals using weighted quantile sum regression, quantile g-computation, Bayesian kernel machine regression and similar mixture approaches, including those that assessed selenium in combination with lead, cadmium, mercury, manganese and other elements (87, 88). These approaches can characterize joint, nonlinear, and potentially interactive associations, but their interpretation remains sensitive to correlated exposure sources, socioeconomic and behavioral confounding, exposure timing, measurement error, and differences among statistical models. Prospective studies with repeated exposure measurements and prespecified mixture analyses are therefore needed to clarify how selenium status modifies, or is modified by, concurrent metal exposure.

Nano-selenium represents a formulation-specific field rather than a single interchangeable intervention category. Chemically reduced elemental particles, template-engineered nanoparticles, biologically or green-synthesized particles, and polymer-stabilized or surface-functionalized particles may possess different size distributions, morphologies, surface chemistries, charges, colloidal stability, protein-corona formation, cellular uptake, selenium-release kinetics, pharmacokinetics, biodistribution, and toxicity (89, 90, 117). Consequently, nano-selenium should not be treated as equivalent to food-derived selenium, selenomethionine, sodium selenite, or other conventional selenium formulations.

Cardiovascular evidence for nano-selenium remains predominantly experimental. Selenium incorporated into guar-gum nanoparticles provided greater protection than non-nanoparticulate selenium in an H9c2 cardiomyoblast ischemia–reperfusion model, with reported effects on oxidative stress, mitochondrial function, apoptosis, and calcium homeostasis (91). More recently, intravenously administered template-synthesized spherical selenium nanoparticles accumulated in cardiac tissue and cardiomyocyte mitochondria in an experimental myocardial ischemia–reperfusion model. Treatment led to suppression of STAT1-related oxidative and inflammatory signaling, preservation of mitochondrial respiration and improvements in experimental cardiac injury and function (92). These studies support formulation-specific mechanistic investigation, while the relevance of their administration routes, doses, tissue distribution, and outcomes to human cardiovascular disease remains to be determined.

Safety is likewise formulation-dependent. Selenium nanoparticles caused concentration-dependent mortality, pericardial oedema, altered heart rate and cardiac arrhythmia in zebrafish embryos with toxicity patterns that were different from sodium selenite (93). The protein-mediated green synthesis has also produced relatively large particles which caused pericardial oedema, reduced heart rate and developmental abnormalities at higher concentrations (94). *In vitro* studies comparing similarly sized particles stabilized with neutral, positively charged or negatively charged polymers showed significant differences in cellular toxicity depending on the surface functionalization (89). Thus, neither nanoscale size nor green synthesis alone is sufficient to predict biocompatibility.

Future cardiovascular nano-selenium studies should report primary and hydrodynamic particle size, size distribution and polydispersity, morphology, elemental and crystalline state, coating composition, surface charge, aggregation and storage stability, dissolution and selenium-release kinetics, residual synthesis reagents, sterility, endotoxin status, and batch reproducibility. *In vivo* studies should additionally characterize route of administration, dose equivalence, pharmacokinetics, organ distribution, myocardial target engagement, clearance, and acute and chronic toxicity. Comparative experiments should use selenium-matched doses and appropriate controls for the selenium precursor, coating material, released ionic selenium, and non-nanoparticulate formulation. These details are necessary because changes in synthesis and surface characteristics can create biologically distinct products even when they share the label “nano-selenium.”

Machine-learning methods have also begun to appear in studies integrating selenium or broader trace-element profiles with cardiovascular phenotypes. In a case–control study of 489 patients with coronary artery disease and 75 controls, 18 trace elements, including selenium, were evaluated alongside lipoprotein subfractions using six diagnostic algorithms; XGBoost showed the strongest overall discrimination. However, selenium was not one of the key differentiating biomarkers highlighted in the report, showing that inclusion as an input variable does not mean necessarily independent predictive importance (95).

In an additional analysis of 6,969 participants from the NHANES, restricted cubic splines and a random-forest model exhibited a U-shaped association between serum selenium and self-reported heart failure, with the lowest estimated prevalence at approximately 150–160 μg/L. The random-forest model exhibited high discrimination within the analyzed dataset, but the study was cross-sectional, used prevalent self-reported heart failure, relied on total serum selenium, and did not provide independent external validation. The estimated range should therefore be considered more appropriately as a finding specific to the population for further evaluation rather than an intervention target that can be directly transferred (96).

Studies aiming to use machine-learning for cardiovascular risk assessment or precision prevention should pre-specify the intended clinical use, separate model development from independent evaluation, avoid information leakage, and report both discrimination and calibration. Analyses should also address missing data and class imbalance. Model performance should be compared to conventional clinical models, and evaluated for temporal and geographic transportability and for incremental and decision-relevant value. Particularly critical in the use of nonlinear algorithms with cross-sectional exposure data or poorly defined cardiovascular outcomes is transparent reporting and structured risk-of-bias assessment (97, 98).

Overall, these emerging themes suggest more integration of environmental exposure science, material characterization, and computational modelling in selenium–cardiovascular research. Their utility will be defined by rigorous exposure metrics, formulation-specific evaluation, reproducible analytical methods, independent validation, and outcomes relevant to cardiovascular biology and patient care.

### 4.9 Geographic context and collaboration priorities

The concentration of research output in China, the United States, and parts of Europe and Scandinavia describes the geographic distribution of scientific activity rather than the distribution of selenium deficiency or cardiovascular disease burden. Because national selenium intake or status, cardiovascular disease burden, research expenditure, and workforce capacity were not included as explanatory covariates, the present analysis cannot determine why publication output differed among countries. Selenium in the food chain varies greatly from region to region and environmental change may affect future availability (12–15, 99). Thus, data from selenium-replete, supplement-using populations may have limited applicability to low-intake areas and severe-deficiency settings should not be used to justify supplementation in other areas.

Future international collaboration should extend beyond co-authorship counts toward harmonized multiregional research protocols. Such protocols should include standardized sampling procedures, calibrated selenium and selenoprotein assays, dietary assessment, inflammatory and renal markers, and adjudicated cardiovascular outcomes. Biobanks in under-represented and potentially selenium-vulnerable regions would be particularly valuable. These designs could directly test whether geographic differences in cardiovascular associations are related to selenium intake, biomarker distributions, genetics, comorbidity, food systems, or health-care access.

### 4.10 A testable precision-prevention agenda

The next phase of research should move beyond asking whether selenium supplementation prevents cardiovascular disease in unselected populations and instead evaluate a staged program of biomarker-enriched interventions. Symptomatic chronic heart failure provides a defensible initial setting because low circulating selenium has been associated with poorer exercise capacity, lower health-related quality of life, and adverse outcomes (36, 100). Other cardiovascular phenotypes, including coronary artery disease, stroke, hypertension, and perioperative myocardial injury, should initially be investigated separately because their underlying mechanisms, selenium-status distributions, and clinically relevant endpoints differ.

A pragmatic first step would be a multicenter, randomized, double-blind, placebo-controlled phase IIb trial in patients with stable symptomatic heart failure receiving guideline-directed medical therapy. The initial trial should prespecify a single heart-failure phenotype, such as heart failure with reduced ejection fraction, rather than combining different ejection-fraction categories without adequate stratification. Randomization could be stratified by study region and baseline selenium status, with renal function and a limited set of established prognostic variables incorporated into the prespecified analysis plan. A dose-ranging design or biomarker-guided titration strategy would be preferable to a single empiric high dose because selenium responses are likely to depend on baseline status and may follow a nonlinear benefit–risk relationship (101). The mechanistic framework in Figure 9 and the selenium-status model in Figure 10 provide the biological and conceptual rationale for this staged program.

Eligibility should require confirmation of low selenium status in two samples collected under standardized conditions during a clinically stable period. A serum selenium concentration below 70 μg/L could be evaluated as a conservative enrichment criterion because this threshold has been associated with poorer functional capacity, quality of life, and prognosis in patients with heart failure and was used in a previous proof-of-concept intervention (36, 102). However, the peripartum-cardiomyopathy trial was open-label and did not demonstrate a statistically significant reduction in its primary composite outcome; the threshold should therefore be regarded as an eligibility hypothesis rather than a predictor of treatment benefit.

Evidence of functional insufficiency could additionally include SELENOP below an assay-specific lower reference boundary or lower population quantile. Because measured SELENOP concentrations vary according to assay design and calibration, the same validated analytical method should be used at enrolment and throughout follow-up (65, 66, 68). Plasma extracellular GPx activity below a validated laboratory-specific lower reference limit may serve as a supportive biomarker, but it should not initially be used as a universal entry criterion because enzyme-activity assays differ in substrates, reaction conditions, calibration, and reporting units (60, 69).

Inflammatory markers, serum albumin, hepatic and renal function, thyroid and iron status, and volume status should be assessed concurrently. These measurements would distinguish potentially selenium-responsive insufficiency from low selenium or selenoprotein concentrations that are mainly related to inflammation, malnutrition, impaired protein synthesis, renal dysfunction, hemodilution, or advanced disease severity (34, 100).

A treatment period of approximately 24–36 weeks would allow assessment of biochemical target engagement and intermediate clinical response. The primary phase II mechanistic endpoint could be the change in log-transformed NT-proBNP from baseline to 24 weeks, analyzed with adjustment for the baseline value and prespecified randomization factors. Key secondary endpoints should include changes in the Kansas City Cardiomyopathy Questionnaire score, 6-min walk distance, peak oxygen consumption where feasible, NYHA functional class, and echocardiographic measures such as left ventricular ejection fraction and global longitudinal strain. Biomarkers, functional capacity, symptoms and patient-reported health status are often combined in heart-failure trials as these outcomes measure different aspects of treatment response (103, 104).

Because exercise-based outcomes can be influenced by frailty, musculoskeletal disease, motivation, learning effects, and repeated-test variability, testing procedures should be standardized and duplicate baseline assessments could be considered. Serum selenium, SELENOP, and plasma extracellular GPx or GPX3 activity should be measured serially as pharmacodynamic indicators alongside patient-reported and functional outcomes rather than as substitutes for them.

Biochemical target engagement should be defined prospectively as a change exceeding assay-specific analytical imprecision and expected within-person variation, together with movement toward the assay-specific reference range or evidence of a biomarker-response plateau. An achieved serum selenium concentration of approximately 100–125 μg/L could be explored as an on-treatment range. Concentrations less than 100 μg/L have been defined as low selenium status in worsening heart failure, while selenoprotein responses may approach a plateau at a plasma selenium concentration of about 125 μg/L (100, 101). These estimates were derived from different populations and should not be applied as a fixed cardiovascular target across assays, regions, disease phenotypes, or selenium formulations.

Dose adjustment should therefore be guided by serial biomarker responses rather than by an isolated concentration. Dose escalation should stop once prespecified target engagement or a biomarker-response plateau is reached. Safety monitoring should include adverse events, renal and hepatic indices, thyroid function, fasting glucose or glycated hemoglobin, and clinical manifestations compatible with selenium excess. The selenium formulation, administered dose, adherence, achieved biomarker concentrations, concomitant supplement use, and total estimated exposure should be recorded systematically. The increased long-term mortality observed with 300 μg/day in the Denmark PRECISE trial further argues against empiric high-dose escalation (101).

Progression to a phase III outcome trial would be reasonable only if the phase II study demonstrated biochemical target engagement accompanied by directionally concordant improvement in NT-proBNP and at least one functional or patient-reported endpoint. The confirmatory trial should be multicenter, event-driven, randomized, double-blind, and placebo-controlled. Its primary endpoint could be the time to first occurrence of cardiovascular death or worsening heart failure, defined as hospitalization for heart failure or an urgent heart-failure visit requiring intravenous therapy. Similar composite endpoints have been used in recent heart-failure outcome trials (105, 106).

The primary analysis should be by intention-to-treat, using a prespecified Cox proportional-hazards model stratified by the randomization factors and with covariate adjustment limited to a small number of known baseline prognostic variables. The proportional-hazards assumption needs to be tested rather than assumed. If substantial non-proportionality is anticipated, alternative analyses based on time-varying treatment effects or restricted mean survival time should be prespecified.

Key secondary outcomes should include total first and recurrent heart-failure events, cardiovascular mortality, all-cause mortality, KCCQ score, and functional capacity. Recurrent events should be analyzed using a prespecified method that accounts for dependence among repeated events and death as a terminal event, such as a joint frailty model or another appropriately justified recurrent-event method (107). Clinical events should be classified using prespecified definitions and adjudicated by a committee blinded to treatment allocation, particularly when distinguishing heart-failure hospitalization, urgent outpatient treatment, and non-heart-failure admission (104).

Exploratory mediation analyses could examine whether changes in SELENOP, extracellular GPx/GPX3 activity, or total selenium account for part of any observed treatment effect. These analyses should be prespecified and interpreted alongside clinical outcomes because a pharmacodynamic biomarker response would establish biological engagement but would not by itself demonstrate cardiovascular benefit.

This staged program would first determine whether correction of documented selenium insufficiency modifies functional selenium biology and improves clinically relevant intermediate outcomes before exposing a larger population to prolonged supplementation. It would also allow eligibility thresholds, biomarker-response criteria, dose-selection rules, and on-treatment ranges to be refined prospectively before broader clinical application.

## 5 Strengths and limitations

The study analyzed temporal, collaborative, journal, reference, and keyword structures through a combination of four bibliographic databases, explicit screening, and complementary software. The narrative element was built around clinically relevant differences in intake, total status, functional biomarkers, supplementation and cardiovascular endpoints. This approach ensured that bibliometric results raised questions without considering the number of publications or citation bursts as signs of treatment effectiveness.

There are some limitations to keep in mind. The included corpus and network structure can be affected by database coverage, indexing practice, language restrictions and the quality of metadata. Citation indicators are biased towards older papers and are influenced by field size, self-citations and reference matching in the database. Notably, our analyses did not exclude self-citations, meaning the 2024–2026 frontier signals may blend broad disciplinary consensus with specific teams’ internal momentum. Future comparative topological analyses should evaluate how excluding self-citations affects these frontier signals. The 2026 output is not a complete annual count, but only records available through May 12. The choices of keyword merging, threshold selection, clustering algorithms and visualization decisions require analytic judgement. However, a key strength of this study is the sensitivity analysis, which validates the robustness of the constructed bibliometric works and parameter selection. The purpose of the narrative synthesis was to interpret the themes that were mapped rather than to estimate pooled clinical effects, and therefore it did not include a formal risk-of-bias assessment or GRADE evaluation. The preliminary BIBLIO reporting guideline is a useful benchmark for the transparency of bibliometric reviews in biomedical research, but its authors themselves acknowledge that more assessment is required (108).

Finally, being bibliometrically prominent is not equivalent to evidentiary strength. A highly cited reference may be influential as it framed a controversy, introduced a method or served as a broad review. A newer prospective cohort or randomized trial may be clinically more informative, despite fewer citations. Therefore, the present review should complement rather than replace endpoint-specific systematic reviews, causal analyses and trials.

## 6 Conclusion

Over the past two decades, selenium-CVD research has evolved from deficiency-related cardiomyopathy and antioxidant biology to functional biomarkers, heart failure phenotyping, ferroptosis, environmental interactions, nano-selenium, and precision-oriented prevention. The bibliometric landscape documents this evolution, but it also reveals a field in which mechanistic plausibility, observational associations, and supplementation evidence have not yet converged.

There is no current evidence to support a universal selenium supplement for cardiovascular disease. Alternatively, selenium status may be used as a marker of nutritional or functional vulnerability in selected populations. Provided intake, total circulating status, functional selenoprotein measures, cardiovascular phenotype, regional context, dose and safety are distinguished. The most credible way forward is status-stratified, biomarker-verified, endpoint-specific intervention research. Until such trials demonstrate clinical benefit, precision prevention should be a research paradigm rather than a blanket recommendation for supplementation.

## Data Availability

Publicly available datasets were analyzed in this study. This data can be found at: the Web of Science Core Collection (WoSCC, https://www.webofscience.com/), Scopus (https://www.scopus.com/), PubMed (https://pubmed.ncbi.nlm.nih.gov/), and Embase (https://www.embase.com/). The detailed search strategies applied to these databases are documented in the Methods section of this article.
