# Supplementary material for: Selenium nutritional status and cardiovascular diseases: mechanisms of cardioprotection and 21 years of global research trends in precision prevention (2005–2026)
Source: Front Nutr. 2026 Aug 31;13:1920059. doi: 10.3389/fnut.2026.1920059 (PMC13572298; doi:10.3389/fnut.2026.1920059)
Supplement: Supplementary file 1 [file Supplementary_file_1.DOCX]

**Supplementary Material**

**Title:** **Selenium nutritional status and cardiovascular diseases: mechanisms of cardioprotection and twenty-one years of global research trends in precision prevention (2005–2026)**

**Authors:** Hao Chen^†^, Shenghui Jin^†^, Xinyi Yuan, Xuyang Zhong, Junqi Xiao, Jiayou Liao^*^, Xiangtai Zeng^*^

**Contents:**

**Supplementary Figure S1:** Sensitivity analysis of the VOSviewer journal co-citation network. (Minimum number of citations of a source: 10, 30, 20).

**Supplementary Figure S2:** Sensitivity analysis of the VOSviewer keyword co-occurrence network. (Minimum number of occurrences of a keyword: 3, 10, 5).

**Supplementary Figure S3:** Sensitivity analysis of the top 25 references with the strongest citation bursts (g-index = 15, 35, and 25).

**Supplementary Figure S4:** Sensitivity analysis of the CiteSpace author keyword clustering under varying g-index scale factors (g-index = 15, 35, and 25).

**Supplementary Figure S5:** Sensitivity analysis of the CiteSpace author keyword clustering under varying maximum links per node (L/N = 5, 15, and 10).

**Supplementary Figure S6:** Sensitivity analysis of the CiteSpace author keyword clustering under varying link retaining factors (LRF = 2.0, 3.0, and 2.5).

**Supplementary Figure S7:** Sensitivity analysis of the top 25 author keywords with the strongest citation bursts under varying g-index scale factors (g-index = 15, 35, and 25).

**Supplementary Figure S8:** Sensitivity analysis of the top 25 author keywords with the strongest citation bursts under varying maximum links per node (L/N = 5, 15, and 10).

**Supplementary Figure S9:** Sensitivity analysis of the top 25 author keywords with the strongest citation bursts under varying link retaining factors (LRF = 2.0, 3.0, and 2.5).

**Figure S1:** Sensitivity analysis of the VOSviewer journal co-citation network under varying minimum citations of a source. (A) Minimum number of citations = 10; (B) Minimum number of citations = 30; (C) Minimum number of citations = 20 (Baseline). The green cluster (represented by *Lancet*, *J Nutr*, and *Am J Clin Nutr*), the red cluster (represented by *Nutrients* and *Biol Trace Elem Res*), and the orange cluster (represented by *J Biol Chem* and *Free Radical Bio Med*) maintained their grouping patterns, and the principal nodes did not change significantly. As shown in (A), lowering the threshold caused the clusters to interlock with each other, making the network visually cluttered and difficult to interpret. Conversely, when the threshold was raised (B), the retained core journals and thematic clusters remained highly consistent with the baseline original figure (C). This suggests that the identified journal co-citation structure demonstrates high robustness.

**Figure S2:** Sensitivity analysis of the VOSviewer keyword co-occurrence network under varying minimum occurrence thresholds. (A) Minimum number of occurrences = 3; (B) Minimum number of occurrences = 10; (C) Minimum number of occurrences = 5 (Baseline). The main keyword nodes and core clustering structure of the network demonstrated good stability across different thresholds. Key thematic nodes such as *selenium*, *oxidative stress*, *cardiovascular disease*, and *selenoproteins* maintained their prominent positions and cluster grouping patterns. As shown in (A), lowering the threshold introduced a large number of peripheral keywords, causing the clusters to interlock and making the network visually cluttered and difficult to interpret. Conversely, when the threshold was raised (B), the retained core keywords and primary thematic clusters remained highly consistent with the baseline original figure (C). This suggests that the identified keyword co-occurrence structure demonstrates high robustness.

**Figure S3:** Sensitivity analysis of the top 25 references with the strongest citation bursts under varying g-index scale factors**.** (A) g-index = 15; (B) g-index = 35; (C) g-index = 25 (Baseline). Highly impactful core references, such as Flores-Mateo G (2006), Stranges S (2007), and Bleys J (2007), consistently appeared at the top of the burst lists across all three conditions. As shown in (A), lowering the g-index shared 19 core burst references with the baseline (C). When the g-index was raised (B), the resulting list retained 21 overlapping references, remaining highly consistent with the baseline. This suggests that the identification of key emergent literature in this field demonstrates high robustness.

**Figure S4:** Sensitivity analysis of the CiteSpace author keyword clustering under varying g-index scale factors. (A) g-index = 15 (Q = 0.3758, S = 0.7337); (B) g-index = 35 (Q = 0.4972, S = 0.8132); (C) g-index = 25 (Baseline; Q = 0.4631, S = 0.7859).

The core research theme of *cardiovascular disease* consistently appeared as a major cluster across all conditions (Clusters #0 and #5 in A; Cluster #0 in B and C). As shown in (A), lowering the g-index resulted in a sparser network with fewer nodes, but it successfully retained major baseline (C) clusters such as *Keshan disease* and *trace elements*. Conversely, raising the g-index (B) incorporated more nodes and generated more granular sub-clusters, while still retaining key overlapping themes with the baseline, such as *trace elements, selenium nanoparticles* and *cardiovascular disease*. This suggests that the main clustering themes are highly robust against g-index variations.

**Figure S5:** Sensitivity analysis of the CiteSpace author keyword clustering under varying maximum links per node (L/N). (A) L/N = 5; (B) L/N = 15; (C) L/N = 10 (Baseline). Across all three settings, the Q and S values remained identical at 0.4631 and 0.7859, respectively. The total number of clusters and their specific labels were also unaffected. This indicates that no keyword formed more than 5 connections per time slice under the set g-index. Consequently, no network edges were further pruned. This confirms that the baseline clustering results are highly robust and insensitive to L/N parameter variations.

**Figure S6:** Sensitivity analysis of the CiteSpace author keyword clustering under varying link retaining factors (LRF). (A) LRF = 2.0 (Q = 0.4631, S = 0.7859); (B) LRF = 3.0 (Q = 0.4572, S = 0.7825); (C) LRF = 2.5 (Baseline; Q = 0.4631, S = 0.7859). The top core research themes, such as *cardiovascular disease*, *selenium nanoparticles*, and *trace elements*, consistently appeared as the major clusters across all conditions. When the LRF was lowered to 2.0, the network structure and evaluation metrics remained identical to the baseline, indicating that the baseline setting (LRF = 2.5) has already retained the stable and core network connections. Conversely, raising the LRF (B) incorporated more weakly correlated links, introducing inter-cluster noise and thus leading to a slight decrease in Q and S values. This further supports the robustness of the baseline parameters.

**Figure S7:** Sensitivity analysis of the top 25 author keywords with the strongest citation bursts under varying g-index scale factors. (A) g-index = 15; (B) g-index = 35; (C) g-index = 25 (Baseline). Highly ranked keywords such as *glutathione peroxidase activity*, *alpha tocopherol*, *trace element*, and *Keshan disease* maintained their prominent positions. As shown in (A), lowering the g-index shared 20 core burst keywords with the baseline (C). When the g-index was raised (B), the resulting list retained 22 overlapping keywords, remaining highly consistent with the baseline. This suggests that the identification of key emergent research topics demonstrates high robustness.

**Figure S8:** Sensitivity analysis of the top 25 author keywords with the strongest citation bursts under varying maximum links per node (L/N). (A) L/N = 5; (B) L/N = 15; (C) L/N = 10 (Baseline). As shown in both (A) and (B), lowering or raising the L/N threshold did not alter the composition of the burst keyword list, retaining all 25 core keywords identical to the baseline (C). The consistent results suggest that citation bursts are insensitive to the L/N parameter.

**Figure S9:** Sensitivity analysis of the top 25 author keywords with the strongest citation bursts under varying link retaining factors (LRF). (A) LRF = 2.0; (B) LRF = 3.0; (C) LRF = 2.5 (Baseline). Similarly, lowering or raising the LRF threshold did not alter the composition of the burst keyword list, retaining all 25 core keywords identical to the baseline (C). The consistent results suggest that citation bursts are insensitive to the LRF parameter.
