# Supplementary material for: Selenium nutritional status and cardiovascular diseases: mechanisms of cardioprotection and 21 years of global research trends in precision prevention (2005–2026)
Source: Front Nutr. 2026 Aug 31;13:1920059. doi: 10.3389/fnut.2026.1920059 (PMC13572298; doi:10.3389/fnut.2026.1920059)
Supplement: Supplementary file 2 [file Table_1.docx]

Web of Science Core Collection Search Strategy:

(TI=("cardiovascular*" OR "cardiac*" OR "cardio*" OR "heart*" OR "myocardial*" OR "pericardial*" OR "arrhythmia*" OR "artery disease*" OR "hypertension") OR AB=("cardiovascular*" OR "cardiac*" OR "cardio*" OR "heart*" OR "myocardial*" OR "pericardial*" OR "arrhythmia*" OR "artery disease*" OR "hypertension") OR AK=("cardiovascular*" OR "cardiac*" OR "cardio*" OR "heart*" OR "myocardial*" OR "pericardial*" OR "arrhythmia*" OR "artery disease*" OR "hypertension"))

AND (TI="Selenium" OR AB="Selenium" OR AK="Selenium")

AND Publication Data=(2005-01-01) to (2026-05-12)

AND Document Type=(Article OR Review)

AND Language=(English)

Scopus Search Strategy:

( TITLE-ABS ( "cardiovascular*" OR "cardiac*" OR "cardio*" OR "heart*" OR "myocardial*" OR "pericardial*" OR "arrhythmia*" OR "artery disease*" OR "hypertension" ) OR AUTHKEY ( "cardiovascular*" OR "cardiac*" OR "cardio*" OR "heart*" OR "myocardial*" OR "pericardial*" OR "arrhythmia*" OR "artery disease*" OR "hypertension" ) )

AND ( TITLE-ABS ( "selenium" ) OR AUTHKEY ( "selenium" ) )

AND PUBYEAR > 2004 AND PUBYEAR < 2027

AND ( LIMIT-TO ( DOCTYPE , "ar" ) OR LIMIT-TO ( DOCTYPE , "re" ) )

AND ( LIMIT-TO ( LANGUAGE , "English" ) )

PubMed Search Strategy:

( "cardiovascular*"[Title/Abstract] OR "cardiac*"[Title/Abstract] OR "cardio*"[Title/Abstract] OR "heart*"[Title/Abstract] OR "myocardial*"[Title/Abstract] OR "pericardial*"[Title/Abstract] OR "arrhythmia*"[Title/Abstract] OR "artery disease*"[Title/Abstract] OR "hypertension"[Title/Abstract] )

AND ( "selenium"[Title/Abstract] ))

AND ("2005/01/01"[dp] : "2026/05/12"[dp])

AND ("Adaptive Clinical Trial"[pt] OR "Clinical Trial"[pt] OR "Clinical Trial, Phase I"[pt] OR "Clinical Trial, Phase II"[pt] OR "Clinical Trial, Phase III"[pt] OR "Clinical Trial, Phase IV"[pt] OR "Clinical Trial, Veterinary"[pt] OR "Controlled Clinical Trial"[pt] OR "Equivalence Trial"[pt] OR "Multicenter Study"[pt] OR "Pragmatic Clinical Trial"[pt] OR "Randomized Controlled Trial"[pt] OR "Randomized Controlled Trial, Veterinary"[pt] OR "Case Reports"[pt] OR "Clinical Study"[pt] OR "Comparative Study"[pt] OR "Evaluation Study"[pt] OR "Observational Study"[pt] OR "Observational Study, Veterinary"[pt] OR "Twin Study"[pt] OR "Validation Study"[pt] OR "Review"[pt] OR "Systematic Review"[pt] OR "Meta-Analysis"[pt])

AND "English"[la]

Embase Search Strategy:

('cardiovascular*' OR 'cardiac*' OR 'cardio*' OR 'heart*' OR 'myocardial*' OR 'pericardial*' OR 'arrhythmia*' OR 'artery disease*' OR 'hypertension'):ti,ab,kw

AND 'selenium':ti,ab,kw

AND [2005-2026]/py

AND ('article'/it OR 'review'/it)

AND [english]/lim
