# Supplementary material for: Selenium nutritional status and cardiovascular diseases: mechanisms of cardioprotection and 21 years of global research trends in precision prevention (2005–2026)
Source: Front Nutr. 2026 Aug 31;13:1920059. doi: 10.3389/fnut.2026.1920059 (PMC13572298; doi:10.3389/fnut.2026.1920059)
Supplement: Supplementary file 3 [file Table_2.DOCX]

label,replace by

taiwan,china

peoples r china,china

england,united kingdom

north ireland,united kingdom

scotland,united kingdom

united states,usa

turkiye,turkey
