# Supplementary material for: Selenium nutritional status and cardiovascular diseases: mechanisms of cardioprotection and 21 years of global research trends in precision prevention (2005–2026)
Source: Front Nutr. 2026 Aug 31;13:1920059. doi: 10.3389/fnut.2026.1920059 (PMC13572298; doi:10.3389/fnut.2026.1920059)
Supplement: Supplementary file 4 [file Table_3.DOCX]

label,replace by

charite,charite univ med berlin

harvard med sch,harvard univ

harvard th chan sch publ hlth,harvard univ

johns hopkins bloomberg sch publ hlth,johns hopkins univ

johns hopkins med inst,johns hopkins univ

univ hawaii manoa,univ hawaii

hedmark univ coll,inland norway univ appl sci
