# Supplementary material for: Selenium nutritional status and cardiovascular diseases: mechanisms of cardioprotection and 21 years of global research trends in precision prevention (2005–2026)
Source: Front Nutr. 2026 Aug 31;13:1920059. doi: 10.3389/fnut.2026.1920059 (PMC13572298; doi:10.3389/fnut.2026.1920059)
Supplement: Supplementary file 5 [file Table_4.DOCX]

label	replace by

antioxidant	antioxidants

biomarker	biomarkers

cardiovascular diseases	cardiovascular disease

cvd	cardiovascular disease

coenzyme q(10)	coenzyme q10

diabetes	diabetes mellitus

micronutrient	micronutrients

rat	rats

ros	reactive oxygen species

risk factor	risk factors

se	selenium

selenoprotein	selenoproteins

trace element	trace elements

supplements	dietary supplements

supplementation	dietary supplements

congestive heart failure	heart failure

chronic heart failure	heart failure
