# Supplementary material for: Selenium nutritional status and cardiovascular diseases: mechanisms of cardioprotection and 21 years of global research trends in precision prevention (2005–2026)
Source: Front Nutr. 2026 Aug 31;13:1920059. doi: 10.3389/fnut.2026.1920059 (PMC13572298; doi:10.3389/fnut.2026.1920059)
Supplement: Supplementary file 6 [file Table_5.DOCX]

cardiovascular diseases	cardiovascular disease

vascular disease	cardiovascular disease

coronary artery disease	coronary heart disease

ischemic heart disease	coronary heart disease

coronary atherosclerosis	coronary heart disease

acute myocardial infarction	myocardial infarction

chronic heart failure	heart failure

congestive heart failure	heart failure

oxidant stress	oxidative stress

antioxidants	antioxidant

dietary antioxidant index	antioxidant

antioxidant status	antioxidant

ascorbic acid	vitamin c

alpha tocopherol	vitamin e

c reactive protein	c-reactive protein

high-sensitivity c-reactive protein	c-reactive protein

systolic blood pressure	blood pressure

diastolic blood pressure	blood pressure

acquired immune deficiency syndrome	acquired immunodeficiency syndrome

acute heart infarction	myocardial infarction

heart infarction	myocardial infarction

acute renal failure	acute kidney injury

chronic kidney failure	chronic kidney disease

all cause	all-cause mortality

all cause mortality	all-cause mortality

antioxidant activity	antioxidant

antioxidant barrier	antioxidant

antioxidant biomarkers	antioxidant

antioxidant defense	antioxidant

antioxidant potential	antioxidant

alpha lipoic acid	alpha-lipoic acid

coenzyme q(10)	coenzyme q10

20-hydroxyeicosatetraenoic acids	20 hydroxyicosatetraenoic acid

3 untranslated region	3' untranslated region

lipid profile	lipid profiles

nano selenium	selenium nanoparticles

electrocardiogram	electrocardiography

abnormal electrocardiograms	electrocardiography

alpha tocopherol supplementation	vitamin e

vitamin e supplementation	vitamin e

dietary selenium	selenium

selenium intake	selenium

selenium supplementation	selenium

plasma selenium	selenium blood level

serum selenium	selenium blood level

selenium levels	selenium blood level

glutathione peroxidase 1	glutathione peroxidase

glutathione peroxidase 2	glutathione peroxidase

glutathione peroxidase 3	glutathione peroxidase

glutathione peroxidase activity	glutathione peroxidase

thioredoxin reductase 1	thioredoxin reductase

thioredoxin reductase 2	thioredoxin reductase

thioredoxin reductase 3	thioredoxin reductase

arterial hypertension	hypertension

essential hypertension	hypertension
