# Supplementary material for: Selenium nutritional status and cardiovascular diseases: mechanisms of cardioprotection and 21 years of global research trends in precision prevention (2005–2026)
Source: Front Nutr. 2026 Aug 31;13:1920059. doi: 10.3389/fnut.2026.1920059 (PMC13572298; doi:10.3389/fnut.2026.1920059)
Supplement: Supplementary file 7 [file Table_6.DOCX]

@PHRASEarticle

@PHRASEreview

@PHRASEpriority journal

@PHRASEclinical article

@PHRASEcontrolled study

@PHRASEmajor clinical study

@PHRASEclinical trial

@PHRASErandomized controlled trial

@PHRASEcontrolled trial

@PHRASErandomized trial

@PHRASEdouble blind

@PHRASEdouble blind procedure

@PHRASEtrial

@PHRASEmetaanalysis

@PHRASEsystematic review

@PHRASEcase control study

@PHRASEcohort studies

@PHRASEcross sectional study

@PHRASEcross-section study

@PHRASEepidemiology

@PHRASEhuman

@PHRASEhumans

@PHRASEnonhuman

@PHRASEanimal

@PHRASEanimals

@PHRASEanimalia

@PHRASEmale

@PHRASEfemale

@PHRASEmen

@PHRASEwomen

@PHRASEadult

@PHRASEadults

@PHRASEaged

@PHRASEolder adults

@PHRASEmiddle aged

@PHRASEelderly patients

@PHRASEadolescent

@PHRASEchildren

@PHRASErat

@PHRASErats

@PHRASEmouse

@PHRASEmice

@PHRASEfisher

@PHRASEanimal model

@PHRASEanimal experiment

@PHRASEanimal tissue

@PHRASEanimal cell

@PHRASEdisease

@PHRASEdiseases

@PHRASEstatistical significance

@PHRASEanalysis of covariance

@PHRASEvalidation

@PHRASEinstruments

@PHRASEquestionnaire

@PHRASEchemistry

@PHRASEin vitro

@PHRASEin vitro study

@PHRASEin vivo
