# Supplementary material for: Selenium nutritional status and cardiovascular diseases: mechanisms of cardioprotection and 21 years of global research trends in precision prevention (2005–2026)
Source: Front Nutr. 2026 Aug 31;13:1920059. doi: 10.3389/fnut.2026.1920059 (PMC13572298; doi:10.3389/fnut.2026.1920059)
Supplement: Supplementary file 8 [file Table_7.DOCX]

**Supplementary Table 7. Design-level appraisal of the ten most locally cited documents**

| **No.** | **Study** | **Design and evidence base** | **Selenium measure/intervention** **and cardiovascular outcome(s)** | **Key methodological strengths** | **Key limitations and risk of** **overinterpretation** | **Interpretation for the present** **review** |
| --- | --- | --- | --- | --- | --- | --- |
| 1 | **Flores-Mateo et al., 2006**  *Selenium and coronary heart disease: a meta-analysis*  Am J Clin Nutr; LC = 121  DOI: 10.1093/ajcn/84.4.762 | **Systematic review and meta-analysis:** 25 observational studies (14 cohort and 11 case-control) and 6 randomized trials. | Blood or toenail selenium biomarkers in observational studies; selenium-containing supplements in trials. Outcomes were coronary heart disease events or mortality. | Separated observational and randomized evidence; used study-level quality criteria, random-effects pooling, dose-response analysis, and sensitivity analyses. | Searches were largely limited to MEDLINE and the Cochrane Library. Biomarker matrices, populations, confounder adjustment, and endpoints were heterogeneous. Several case-control studies had limited adjustment; trials were few, generally small, and often combined selenium with other nutrients. | A foundational synthesis showing an inverse observational association but inconclusive randomized evidence. It does not support routine selenium supplementation for cardiovascular prevention. |
| 2 | **Bleys et al., 2008**  *Serum selenium levels and all-cause, cancer, and cardiovascular mortality among US adults*  Arch Intern Med; LC = 83  DOI: 10.1001/archinternmed.2007.74 | **Prospective cohort:** 13,887 NHANES III adults with up to 12 years of mortality follow-up. | Single baseline serum selenium measurement. Outcomes were all-cause, cancer, and cardiovascular mortality. | Large nationally representative sample; extensive covariate adjustment; survey-weighted analyses; restricted-spline models assessed nonlinearity. | Observational design, residual confounding, and reliance on one baseline selenium measurement. Cause-specific mortality was based on mortality linkage. The population was largely selenium-replete, and serum selenium was not significantly associated with cardiovascular mortality. | Important for the nonlinear status-window hypothesis and safety caution, but it provides no evidence that increasing selenium reduces cardiovascular mortality. |
| 3 | **Benstoem et al., 2015**  *Selenium and its supplementation in cardiovascular disease—what do we know?*  Nutrients; LC = 76  DOI: 10.3390/nu7053094 | **Narrative review:** selenium biology, selenoproteins, observational evidence, supplementation, myocardial injury, and cardiac-surgery research. | Multiple exposure constructs, including intake, circulating selenium, selenoprotein biology, and supplementation; multiple cardiovascular settings. | Broad mechanistic and clinical scope; clearly summarized the biological plausibility and major translational controversies present at the time. | No systematic-review protocol, reproducible study-selection process, formal risk-of-bias assessment, or quantitative synthesis. Selective emphasis cannot be excluded, and conclusions depend on heterogeneous evidence types. | Highly influential for conceptual framing, but citation prominence and narrative breadth should not be interpreted as high-certainty evidence of supplementation benefit. |
| 4 | **Bomer et al., 2020**  *Selenium and outcome in heart failure*  Eur J Heart Fail; LC = 59  DOI: 10.1002/ejhf.1644 | **Prospective multinational cohort with mechanistic experiments:** BIOSTAT-CHF patients with worsening heart failure, supplemented by experiments in human cardiomyocytes. | Serum selenium measured by inductively coupled plasma mass spectrometry; deficiency defined as <70 μg/L. Outcomes included all-cause mortality, heart-failure hospitalization, symptoms, exercise capacity, and quality of life. | Prospective clinical phenotyping, standardized selenium measurement, clinically relevant outcomes, multinational recruitment, and complementary mechanistic experiments. | Nonrandomized design cannot exclude residual confounding or reverse causation from disease severity, inflammation, poor intake, or altered metabolism. Selenium was measured at baseline, and the <70 μg/L threshold was not validated as a treatment-selection or supplementation target. | Supports low selenium as a prognostic or vulnerability marker in heart failure. It does not establish causality or demonstrate that supplementation improves outcomes. |
| 5 | **Zhang et al., 2016**  *Selenium status and cardiovascular diseases: meta-analysis of prospective observational studies and randomized controlled trials*  Eur J Clin Nutr; LC = 55  DOI: 10.1038/ejcn.2015.78 | **Meta-analysis:** 16 prospective observational studies and 16 randomized controlled trials, including linear and nonlinear dose-response analyses. | Blood selenium status in observational studies and oral selenium supplementation in trials. Outcomes included cardiovascular incidence, mortality, and related endpoints. | Explicitly separated observational and randomized evidence; used random-effects models and restricted cubic splines; examined achieved biomarker changes after supplementation. | Substantial heterogeneity in biomarkers, baseline status, populations, selenium forms, doses, durations, and cardiovascular endpoints. Aggregate-data dose-response estimates cannot define an individual clinical reference interval or treatment target. | Supports a nonlinear observational association while showing no overall cardiovascular benefit in randomized trials. The reported concentration range should remain hypothesis-generating. |
| 6 | **Stranges et al., 2006**  *Effects of selenium supplementation on cardiovascular disease incidence and mortality: secondary analyses in a randomized clinical trial*  Am J Epidemiol; LC = 51  DOI: 10.1093/aje/kwj097 | **Secondary randomized-trial analysis:** cardiovascular outcomes in 1,004 participants without baseline cardiovascular disease from the placebo-controlled Nutritional Prevention of Cancer Trial. | Selenium 200 μg/day versus placebo, with a mean follow-up of approximately 7.6 years. Outcomes included total cardiovascular disease, myocardial infarction, stroke, and cardiovascular mortality. | Randomized placebo-controlled design; long blinded follow-up; clinically meaningful outcomes; analyses stratified by baseline plasma selenium. | Cardiovascular outcomes were secondary rather than the trial's original primary endpoints. The study was not designed or powered specifically for cardiovascular prevention and did not enrich for selenium deficiency. Participants were selected from a cancer-prevention trial population. | Provides evidence against a universal primary-prevention effect of 200 μg/day selenium in this specific cohort, but does not exclude effects in prospectively selected selenium-deficient groups. |
| 7 | **Laclaustra et al., 2009**  *Serum selenium concentrations and hypertension in the US population*  Circ Cardiovasc Qual Outcomes; LC = 47  DOI: 10.1161/CIRCOUTCOMES.108.831552 | **Cross-sectional study:** 2,638 NHANES 2003-2004 adults aged ≥40 years. | Single serum selenium measurement. Outcomes were measured blood pressure and prevalent hypertension. | Nationally representative sample; standardized selenium assay and blood-pressure measurement; survey weighting; extensive confounder adjustment; spline analysis of nonlinearity. | Cross-sectional design prevents temporal or causal inference. Reverse causation and residual confounding remain possible. Selenium was measured once, and findings arose in a relatively selenium-replete US population. The outcome was prevalent rather than incident hypertension. | Raises concern that higher selenium status may not be uniformly beneficial, but cannot establish that selenium exposure causes hypertension. |
| 8 | **Alehagen et al., 2013**  *Cardiovascular mortality and N-terminal-proBNP reduced after combined selenium and coenzyme Q10 supplementation*  Int J Cardiol; LC = 46  DOI: 10.1016/j.ijcard.2012.04.156 | **Randomized double-blind trial:** 443 community-dwelling Swedish adults aged 70-88 years. | Combined organic selenium yeast (200 μg/day) and coenzyme Q10 (200 mg/day) versus placebo for 48 months. Outcomes included cardiovascular mortality, NT-proBNP, and echocardiographic function. | Randomized double-blind design; placebo control; long intervention and follow-up; intention-to-treat and per-protocol analyses; clinical, biomarker, and imaging outcomes. | Single-centre trial in a geographically and demographically homogeneous elderly Swedish population. Selenium and coenzyme Q10 were administered together, preventing attribution of effects to selenium alone. Generalizability to younger, selenium-replete, or clinically different populations is limited, and independent replication is required. | A clinically interesting signal from a status-specific combined intervention, not evidence for universal selenium supplementation or a selenium-specific treatment effect. |
| 9 | **Kuria et al., 2021**  *Selenium status in the body and cardiovascular disease: a systematic review and meta-analysis*  Crit Rev Food Sci Nutr; LC = 40  DOI: 10.1080/10408398.2020.1803200 | **Systematic review and meta-analysis:** 13 eligible studies, with meta-regression and dose-response analysis; protocol registered in PROSPERO. | Predominantly blood selenium, with some toenail measurements; cardiovascular incidence and mortality across observational studies and randomized evidence. | Searches across multiple bibliographic databases and gray-literature sources; PRISMA/MOOSE-oriented methods; protocol registration; dose-response and publication-bias analyses. | Only a small number of heterogeneous studies were eligible. Designs, specimens, regions, and outcomes differed, and study-level meta-analysis cannot eliminate confounding in the underlying observational evidence. A published correction amended one extracted mortality estimate and the associated figure. | Supports further investigation of status-dependent and nonlinear associations, but does not establish causal benefit or define a supplementation threshold. |
| 10 | **Bleys et al., 2009**  *Serum selenium and peripheral arterial disease: results from NHANES 2003-2004*  Am J Epidemiol; LC = 36  DOI: 10.1093/aje/kwn414 | **Cross-sectional study:** 2,062 NHANES 2003-2004 adults aged ≥40 years. | Single serum selenium measurement. Peripheral arterial disease was defined objectively using an ankle-brachial index <0.90. | Nationally representative sampling; objective vascular outcome; standardized mass-spectrometry assay; broad confounder adjustment; restricted-spline evaluation of nonlinearity. | Cross-sectional design and single exposure measurement preclude temporality. The number of peripheral arterial disease cases was limited, confidence intervals were wide, and fully adjusted associations were not statistically significant. Results arose in a selenium-replete population. | Suggests a possible U-shaped pattern worthy of prospective testing, but provides no causal or therapeutic evidence. |

Abbreviations: CVD, cardiovascular disease; HF, heart failure; LC, local citations within the analyzed corpus; NHANES, National Health and Nutrition Examination Survey; NT-proBNP, N-terminal pro-B-type natriuretic peptide; RCT, randomized controlled trial. Note: The appraisal is descriptive and design-specific. Because the documents span heterogeneous study designs, no single quantitative score was assigned. This table does not replace formal design-specific risk-of-bias assessment using tools appropriate to each study type.
